# Supplementary material for: Non-parametric genetic prediction of complex traits with latent Dirichlet process regression models
Source: Nat Commun. 2017 Sep 6;8:456. doi: 10.1038/s41467-017-00470-2 (PMC5587666; doi:10.1038/s41467-017-00470-2)
Supplement: Supplementary file 1 — PDF SI [file 41467_2017_470_MOESM1_ESM.pdf]

File name: Supplementary Information
Description: Supplementary figures, supplementary tables, supplementary notes and supplementary references.

Supplementary Note: Model and Algorithm Details for DPR

The latent Dirichlet process regression model

We consider the following multiple linear regression model

$$\mathbf{y} = \mathbf{W}\boldsymbol{\alpha} + \mathbf{X}\tilde{\boldsymbol{\beta}} + \boldsymbol{\varepsilon}, \boldsymbol{\varepsilon} \sim N(0, \sigma_e^2 \mathbf{I}_n), \tag{1}$$

where $\mathbf{y}$ is an n-vector of phenotypes measured on n individuals; $\mathbf{W}$ is an n by c matrix of covariates including a column of 1s for the intercept term; $\boldsymbol{\alpha}$ is a c-vector of coefficients; $\mathbf{X}$ is an n by p matrix of genotypes; $\tilde{\boldsymbol{\beta}}$ is the corresponding p-vector of effect sizes; $\boldsymbol{\varepsilon}$ is an n-vector of residual errors where each element is assumed to be independently and identically distributed from a normal distribution with variance σ_e^2. Note that we use $\tilde{\boldsymbol{\beta}}$ here instead of $\boldsymbol{\beta}$ as in the main text for reasons that will become clear shortly.

As explained in the main text, we assign a normal prior $N(0, \sigma^2\sigma_e^2)$ on each element of $\tilde{\boldsymbol{\beta}}$, and we further assign a Dirichlet process prior on the variance parameter σ^2. (Note that different from the main text, we also scale the variance with the error variance σ_e^2 to simply the algorithm.) Integrating out σ^2 induces a Dirichlet process normal mixture prior on $\tilde{\beta}_i$

$$\tilde{\beta}_i \sim \sum_{k=1}^{\infty} \pi_k N(0, (\sigma_k^2 + \sigma_b^2)\sigma_e^2),$$
$$\pi_k = \nu_k \prod_{l=1}^{k-1}(1-\nu_l), \nu_k \sim \mathrm{Beta}(1, \lambda), \tag{2}$$

where $\sigma_k^2 + \sigma_b^2$ (scaled by σ_e^2) is the variance for each normal component. Again, to simply the algorithm, different from the main text, we add a common variance σ_b^2 to each variance component and we set $\sigma_k^2 = 0$ when $k = 1$. We refer to the above model based on equations (1) and (2) as the latent Dirichlet Process Regression (DPR) model. For the hyper-parameters $\boldsymbol{\alpha}$, σ_k^2, σ_b^2, σ_e^2, and λ in the model, we consider the following priors

$$\alpha_j \sim N(0, \sigma_e^2 \sigma_w^2), \sigma_w^2 \to \infty,$$
$$\sigma_k^2 \sim \text{inverse-gamma } (a_{0k}, b_{0k}),$$
$$\sigma_b^2 \sim \text{inverse-gamma } (a_{0b}, b_{0b}), \quad (3)$$
$$\sigma_e^2 \sim \text{inverse-gamma } (a_{0e}, b_{0e}),$$
$$\lambda \sim \text{gamma}(a_{0\lambda}, b_{0\lambda}),$$

where we set a_{0k}, b_{0k}, a_{0b}, b_{0b}, a_{0e}, and b_{0e} in the inverse gamma distributions to be 0.1; we set $a_{0\lambda}$ and $b_{0\lambda}$ in the gamma distribution to be 1 and 0.1; and we use a limiting normal prior for each α_j with the normal variance goes to infinity, since generally there is enough information in the likelihood to overwhelm any reasonable prior assumption for these parameters.

To improve mixing, following[1], we group the effect sizes that correspond to the first normal component with the smallest variance σ_b^2 in equation (2) into a random effects term **u**:

$$\mathbf{u} = \mathbf{X}\mathbf{b} \sim N(0, \sigma_b^2 \sigma_e^2 \mathbf{K}), \quad (4)$$

where $\mathbf{K} = \mathbf{X}\mathbf{X}^T / p$ is the genetic relatedness matrix (GRM)[1,2] computed using centered SNPs. Note that the GRM is typically positive semi-definite with one eigen-value being zero due to genotype centering. We do not need to deal with the zero eigenvalue because our algorithms do not involve the inverse of GRM. This way, the model in equation (1) becomes

$$\mathbf{y} = \mathbf{W}\boldsymbol{\alpha} + \mathbf{X}\boldsymbol{\beta} + \mathbf{u} + \boldsymbol{\varepsilon}, \boldsymbol{\varepsilon} \sim N(0, \sigma_e^2 \mathbf{I}_n), \quad (5)$$

explaining our use of $\tilde{\boldsymbol{\beta}}$ in equation (1). In our notation, $\tilde{\boldsymbol{\beta}} = \boldsymbol{\beta} + \mathbf{b}$. The corresponding prior on each element of **b** is

$$b_i \sim N(0, \sigma_b^2 \sigma_e^2 / p), \quad (6)$$

and the corresponding prior on each element of $\boldsymbol{\beta}$ is

$$\beta_i \sim \pi_1 N(0, 0 \times \sigma_e^2) + \sum_{k=2}^{\infty} \pi_k N(0, \sigma_k^2 \sigma_e^2). \quad (7)$$

We will develop algorithms for fitting the equivalent model defined in equation (5) in the following text. With the fitting algorithm, we can obtain the posterior mean of $\tilde{\boldsymbol{\beta}}$ as the

sum of the posterior mean of $\boldsymbol{\beta}$ and the posterior mean of $\mathbf{b}$. We use the posterior mean of $\tilde{\boldsymbol{\beta}}$ to compute prediction errors.

Difference between DPR and BayesR

Before we proceed further, it is useful to clarify the difference between DPR and the previously proposed method BayesR[3]. While our method is motivated in part by BayesR, DPR is different from BayesR in five important areas. First, BayesR is a sparse model while DPR is a non-sparse model: BayesR assumes that most SNPs have zero effects while DPR assumes that all SNPs have non-zero effects. As a result, BayesR and DPR are expected to perform differently in sparse vs non-sparse settings. Second, BayesR fixes the ratio between the variance parameters from the three non-zero components to be 0.01:0.1:1. In contrast, DPR estimates the variance of all non-zero components from the data at hand. Inferring parameters from the data instead of fixing them to pre-set values is expected to improve prediction performance. Third, BayesR uses a mixture of three normal distributions for the non-zero component, while DPR uses infinitely many normal distributions *a priori*. Using three normals can sometimes fail to capture the complicated effect size distributions encountered in a range of genetic architectures, as is evident in simulations presented in the main text. Fourth, importantly, it is not straightforward to extend BayesR to accommodate a larger number of normal components. Consequently, while the BayesR software allows users to specify an arbitrary number of components, in those analyses, BayesR also requires users to provide the variance component estimates for these components. It is far from trivial to figure out how one should obtain these variance component estimates for BayesR. In contrast, DPR provides a principled way to extend the simple normal model to accommodate a much larger number of normal components, ensuring robust prediction performance across a range of settings. Fifth, as we will show below, we fix the number of normal components in DPR in practice due to computational reasons. As has been previously shown in other settings[11,12], using a small number of components to approximate the Dirichlet process can undermine its performance. Therefore, we do want to acknowledge that the results we present in the main text are likely conservative estimates of DPR's performance. Better approximations to the Dirichlet process may improve DPR's prediction performance further.

MCMC sampling

Here, we describe our Markov Chain Monte Carlo (MCMC) sampling algorithm to obtain the posterior samples from DPR. To facilitate MCMC, for each SNP i, we assign a vector of indicator variables $\gamma_{ik} \in \{0,1\}$ to indicate which normal component β_i comes from. To improve convergence, we integrate out $\mathbf{u}$ in model (5) and then perform Gibbs sampling by using the conditional distributions for each parameter in turn. Specifically, let $\boldsymbol{\theta} = (\boldsymbol{\alpha}, \boldsymbol{\beta}, \sigma_b^2, \sigma_k^2, \nu_k, \gamma_{ik}, \lambda, \sigma_e^2)$ includes all unknown parameters in model (5), our joint log marginal posterior after integrating out $\mathbf{u}$ is

$$\begin{aligned}
\log p(\boldsymbol{\theta} \mid \mathbf{y}) &= \log p(\mathbf{y} \mid \boldsymbol{\alpha}, \boldsymbol{\beta}, \sigma_b^2, \sigma_e^2) + \log p(\boldsymbol{\beta} \mid \boldsymbol{\gamma}, \sigma_k^2, \sigma_e^2) \\
&\quad + \log p(\boldsymbol{\gamma} \mid \nu_k) + \log p(\nu_k \mid \lambda) + \log p(\sigma_k^2 \mid a_{0k}, b_{0k}) + \log p(\sigma_e^2 \mid a_{0e}, b_{0e}) \\
&\quad + \log p(\sigma_b^2 \mid a_{0b}, b_{0b}) + \log p(\lambda \mid a_{0\lambda}, b_{0\lambda}) \\
&= C - \frac{1}{2}\log|\sigma_e^2 \mathbf{H}| - \frac{1}{2\sigma_e^2}(\mathbf{y} - \mathbf{W}\boldsymbol{\alpha} - \mathbf{X}\boldsymbol{\beta})^T \mathbf{H}^{-1}(\mathbf{y} - \mathbf{W}\boldsymbol{\alpha} - \mathbf{X}\boldsymbol{\beta}) \\
&\quad + \sum_i \sum_{k=2}^{\infty} \gamma_{ik}\left(-\frac{1}{2}\log(\sigma_e^2) - \frac{1}{2}\log(\sigma_k^2) - \frac{\beta_{ik}^2}{2\sigma_k^2\sigma_e^2}\right) \\
&\quad + \sum_i \sum_k^{\infty} \gamma_{ik}\left(\log(\nu_k) + \sum_{l=1}^{k-1}\log(1-\nu_l)\right) + \sum_k^{\infty}\left((\lambda-1)\log(1-\nu_k) + \log(\lambda)\right) \\
&\quad - \sum_k^{\infty}(a_{0k}+1)\log(\sigma_k^2) - \sum_k^{\infty} b_{0k}\sigma_k^{-2} - (a_{0e}+1)\log(\sigma_e^2) - b_{0e}\sigma_e^{-2} \\
&\quad - (a_{0b}+1)\log(\sigma_b^2) - b_{0b}\sigma_b^{-2} + (a_{0\lambda}-1)\log(\lambda) - b_{0\lambda}\lambda,
\end{aligned} \tag{8}$$

where $\mathbf{H} = \mathbf{I}_n + \sigma_b^2 \mathbf{K}$ and C is a normalizing constant. To simplify notation, we will ignore all constant terms from now on. Based on the joint posterior, we can derive the conditional posterior distribution for each parameter in turn. When we derive these conditional distributions, we will also ignore the other parameters which these distributions are conditional on to simplify the presentation.

Sampling α_j

First, for α_j we have

$$\log p(\alpha_j \mid .) = -\frac{\sigma_e^{-2}\mathbf{w}_j{}^T\mathbf{H}^{-1}\mathbf{w}_j}{2}\alpha_j^2 + \sigma_e^{-2}\mathbf{w}_j{}^T\mathbf{H}^{-1}(\mathbf{y} - \sum_{m\neq j}\mathbf{w}_m\alpha_m - \mathbf{X}\boldsymbol{\beta})\alpha_j. \tag{9}$$

Therefore, the conditional distribution for sampling α_j is $p(\alpha_j \mid .) = N(m_j, s_j^2)$, where

$$m_j = \frac{\mathbf{w}_j{}^T \mathbf{H}^{-1}(\mathbf{y} - \sum_{m \neq j} \mathbf{w}_m \alpha_m - \mathbf{X}\boldsymbol{\beta})}{\mathbf{w}_j{}^T \mathbf{H}^{-1} \mathbf{w}_j}, \qquad s_j^2 = \frac{\sigma_e^2}{\mathbf{w}_j{}^T \mathbf{H}^{-1} \mathbf{w}_j}. \tag{10}$$

Sampling β_{ik} and γ_{ik}

For β_{ik} and γ_{ik}, we have

$$\begin{aligned} \log p(\beta_{ik}, \gamma_{ik} \mid .) = & -\frac{\sigma_e^{-2} \mathbf{x}_i^T \mathbf{H}^{-1} \mathbf{x}_i}{2} \beta_i^2 + \sigma_e^{-2} \mathbf{x}_i^T \mathbf{H}^{-1}(\mathbf{y} - \mathbf{W}\boldsymbol{\alpha} - \sum_{m \neq i} \mathbf{x}_m \beta_m) \beta_i \\ & + \gamma_{ik}(-\frac{1}{2}\log(\sigma_e^2) - \frac{1}{2}\log(\sigma_k^2) - \frac{1}{2}\sigma_e^{-2}\sigma_k^{-2}\beta_{ik}^2) + \gamma_{ik}(\log(\nu_k) + \sum_{l=1}^{k-1}\log(1-\nu_l)). \end{aligned} \tag{11}$$

Therefore, the conditional distributions for sampling β_{ik} and γ_{ik} are

$$\begin{aligned} & p(\beta_{ik} \mid \gamma_{ik} = 1, .) = N(m_{ik}, s_{ik}^2), \\ & p(\gamma_{ik} = 1 \mid .) = \pi_{ik} \propto e^{m_{ik}^2/2s_{ik}^2 + \log(s_{ik}) - \log(\sigma_e) - \log(\sigma_k) + \log(\nu_k) + \sum_{l=1}^{k-1}\log(1-\nu_l)}, \end{aligned} \tag{12}$$

where

$$m_{ik} = \frac{\mathbf{x}_i^T \mathbf{H}^{-1}(\mathbf{y} - \mathbf{W}\boldsymbol{\alpha} - \sum_{m \neq i} \mathbf{x}_m \beta_m)}{\mathbf{x}_i^T \mathbf{H}^{-1} \mathbf{x}_i + \sigma_k^{-2}}, \qquad s_{ik}^2 = \frac{\sigma_e^2}{\mathbf{x}_i^T \mathbf{H}^{-1} \mathbf{x}_i + \sigma_k^{-2}}. \tag{13}$$

Sampling v_k

For v_k, we have

$$\log p(\nu_k \mid .) = \sum_i \gamma_{ik} \log(\nu_k) + \sum_i \sum_{l=k+1}^{\infty} \gamma_{il} \log(1-\nu_k) + (\lambda - 1)\log(1-\nu_k). \tag{14}$$

Therefore, the conditional distribution for sampling v_k is $p(\nu_k \mid .) = \mathrm{Beta}(\kappa_k, \lambda_k)$, where

$$\kappa_k = \sum_i \gamma_{ik} + 1, \qquad \lambda_k = \sum_i \sum_{l=k+1}^{\infty} \gamma_{il} + \lambda. \tag{15}$$

Sampling $\boldsymbol{\sigma_k^2}$

For σ_k^2, we have

$$\log p(\sigma_k^2 \mid .) = -(\frac{\sum_i \gamma_{ik}}{2} + a_{0k} + 1)\log(\sigma_k^2) - (\frac{\sum_i \gamma_{ik}\beta_{ik}^2 \sigma_e^{-2}}{2} + b_{0k})\sigma_k^{-2}. \quad (16)$$

Therefore, the conditional distribution for sampling σ_k^2 is $p(\sigma_k^2 \mid .) = \text{inverse-gamma}(a_k, b_k)$, where

$$\begin{aligned} a_k &= \frac{1}{2}\sum_i \gamma_{ik} + a_{0k}, \\ b_k &= \frac{1}{2\sigma_e^2}\sum_i \gamma_{ik}\beta_{ik}^2 + b_{0k}. \end{aligned} \quad (17)$$

Sampling $\boldsymbol{\lambda}$

For λ, we have

$$\log p(\lambda|.) = \lambda(\sum_k^{\infty} \log(1-v_k) - b_{0\lambda}) + \log(\lambda)(a_{0\lambda} + \sum_k^{\infty} 1_k). \quad (18)$$

Therefore, the conditional distribution for sampling λ is $p(\lambda|.) = \text{gamma}(a_\lambda, b_\lambda)$, where

$$\begin{aligned} a_\lambda &= a_{0\lambda} + \sum_k^{\infty} 1_k, \\ b_\lambda &= b_{0\lambda} - \sum_k^{\infty} \log(1-v_k). \end{aligned} \quad (19)$$

Sampling $\boldsymbol{\sigma_e^2}$

For σ_e^2, we have

$$\begin{aligned} \log p(\sigma_e^2 \mid .) = &-((n + \sum_i \sum_{k=2} \gamma_{ik})/2 + a_{0e} + 1)\log(\sigma_e^2) - \frac{1}{2}\text{SSR} \times \sigma_e^{-2} \\ &-\frac{1}{2}(\sum_i \sum_k \gamma_{ik}\beta_{ik}^2\sigma_k^{-2} + 2b_{0e})\sigma_e^{-2}. \end{aligned} \quad (20)$$

Therefore, the conditional distribution for sampling σ_e^2 is $p(\sigma_e^2 \mid .)$ = inverse-gamma(a_e, b_e) where

$$a_e = n/2 + \sum_i \sum_{k=2} \gamma_{ik} / 2 + a_{0e},$$
$$b_e = \frac{1}{2}(\mathrm{SSR} + \sum_i \sum_{k=2} \gamma_{ik} \beta_{ik}^2 / \sigma_k^2 + 2b_{0e}), \tag{21}$$
$$\mathrm{SSR} = (\mathbf{y} - \mathbf{W}\boldsymbol{\alpha} - \mathbf{X}\boldsymbol{\beta})^T \mathbf{H}^{-1} (\mathbf{y} - \mathbf{W}\boldsymbol{\alpha} - \mathbf{X}\boldsymbol{\beta}).$$

Sampling $\boldsymbol{\sigma_b^2}$

For σ_b^2, we have

$$\log p(\sigma_b^2 \,|\, .) = -\frac{1}{2}\log|\mathbf{H}| - \frac{1}{2\sigma_e^2}(\mathbf{y} - \mathbf{W}\boldsymbol{\alpha} - \mathbf{X}\boldsymbol{\beta})^T \mathbf{H}^{-1} (\mathbf{y} - \mathbf{W}\boldsymbol{\alpha} - \mathbf{X}\boldsymbol{\beta}) \\ - (a_{0b} + 1)\log(\sigma_b^2) - b_{0b}\sigma_b^{-2}, \tag{22}$$

which is in an unknown distributional form. Nevertheless, it is straightforward to sample from this univariate distribution using reject sampling, importance sampling or other standard methods[4]. Here, we sample σ_b^2 based on re-parameterization of σ_b^2 following[1,5]. Specifically, we define a new parameter (h^2)[2,6,7]

$$h^2 = \frac{\sigma_b^2}{\sigma_b^2 + 1}, \tag{23}$$

which is bounded between 0 and 1. The log-posterior conditional distribution for h^2 is

$$\log p(h^2 \,|\, .) = \log p(\sigma_b^2(h^2) \,|\, .) - 2\log(1 - h^2), \tag{24}$$

where $p(\sigma_b^2(h^2) \,|\, .)$ is the posterior conditional distribution given in (22) with $\sigma_b^2(h^2) = h^2/(1-h^2)$. We then use the Metropolis-Hastings algorithm to generate posterior samples for h^2. In particular, we use the independent random walk algorithm for h^2 with a Beta(2,8) distribution as the proposal distribution. With each sampled value of h^2, we can obtain a sampled value of $\sigma_b^2 = h^2/(1-h^2)$.

Sampling b

Finally, because of the relationship between **u** and **b** in equation (4), we can obtain the posterior conditional distribution for **b** as

$$p(\mathbf{b} \,|\, .) = \mathrm{MVN}_p(\frac{\sigma_b^2}{p}\mathbf{X}^T\mathbf{H}^{-1}(\mathbf{y} - \mathbf{W}\boldsymbol{\alpha} - \mathbf{X}\boldsymbol{\beta}), \sigma_b^2\sigma_e^2(p^{-1}\mathbf{I}_p - p^{-2}\sigma_b^2\mathbf{X}^T\mathbf{H}^{-1}\mathbf{X})), \tag{25}$$

where $MVN_p(\boldsymbol{\mu}, \boldsymbol{\Sigma})$ is a p-dimensional multivariate normal distribution with mean $\boldsymbol{\mu}$ and variance-covariance $\boldsymbol{\Sigma}$. To reduce variance, we use the Rao-Blackwellised approximation to compute the mean of $\mathbf{b}$ at the end of the MCMC sampling, with

$$\hat{\mathbf{b}} = \frac{1}{p}\mathbf{X}^T \frac{1}{L}\sum_{\ell=1}^{L}(\sigma_b^2)^{(\ell)}(\mathbf{H}^{(\ell)})^{-1}(\mathbf{y} - \mathbf{W}\boldsymbol{\alpha}^{(\ell)} - \mathbf{X}\boldsymbol{\beta}^{(\ell)}). \qquad (26)$$

where L is the total iterations of MCMC after burn in, ℓ denotes the posterior samples. These $\hat{\mathbf{b}}$ are added back to the posterior mean of $\boldsymbol{\beta}$ to yield the posterior mean of $\tilde{\boldsymbol{\beta}}$.

Efficient computation

We apply the algebra innovations recently developed for linear mixed models[1,8,9] to improve computational efficiency. Specifically, at the beginning of MCMC, we perform an eigen decomposition of $\mathbf{K} = \mathbf{UDU}^T$, where $\mathbf{U}$ is the matrix of eigenvectors and $\mathbf{D}$ is a diagonal matrix of eigenvalues[1,8,9]. Then we transform phenotype, genotypes and covariates as $\mathbf{U}^T\mathbf{y}$, $\mathbf{U}^T\mathbf{X}$, and $\mathbf{U}^T\mathbf{W}$. Afterwards, the likelihood conditional on the transformed variables become independent, thus alleviating much of the computational burden associated with the complex covariance structure resulted from the random effects $\mathbf{u}$.

The per-iteration computational cost of the above naive MCMC algorithm, after applying the linear mixed model algebra innovations, scales linearly both with the number of individuals and with the number of SNPs. Such computational cost can still be burdensome when we have millions of SNPs. To improve computation efficiency further, we develop a new, prioritized sampling strategy based on the recently developed random scan Gibbs sampler[10,11]. Specifically, we take advantage of the fact that for any complex traits, most SNPs have small effects (or are non-causal) while only a small proportion of SNPs have large effects (or are causal). The likely causal SNPs are important for phenotype prediction and their effect sizes need to be estimated accurately. In contrast, the likely non-causal SNPs often do not influence prediction performance much and their effect sizes individually do not require accurate estimation. Therefore, it is desirable to spend a large amount of computational resource on sampling likely causal SNPs to obtain accurate effect size estimates, while assigning a small amount of resource on sampling likely non-causal SNPs. Certainly, the above arguments are all conditional on a

fixed number of SNPs (i.e. spend extra computational resource on updating a fixed number of likely causal SNPs vs updating a fixed number of likely non-causal SNPs). To perform such prioritized sampling, we first obtain the top M marginally significant SNPs using LMM with the GEMMA algorithm. We treat these M selected SNPs as likely causal SNPs and update their effect sizes in each MCMC iteration. We then treat the unselected SNPs as likely non-causal SNPs and update their effect sizes once every T iterations. We set $M = 500$ and $T = 10$ (both are set to allow fast computation since the association signals are relatively strong in these two data) for the cattle and maize data, $M = 10^5$ and $T = 2$ (the two are set differently as the signals are relatively weak in this data) for the FHS data in the present study; for the GEUVADIS data we performed a full MCMC sampling as the small sample size there allows for efficient computation. Note that the choice M and T theoretically does not affect the stationary distribution, and we recommend exploring a few values of M and T in practice to achieve a balance between speed and accuracy. By prioritizing the computation resource on sampling the likely causal SNPs, our computational algorithm results in a dramatic reduction in computational cost, while yielding the same stationary distribution and maintaining the predictive performance of DPR. As an example, for the three traits MFP, MY and SCS in the cattle data, our naive MCMC takes approximately 25 hours to run 50,000 MCMC iterations. In contrast, our prioritized sampling algorithm reduces the computational cost down to approximately 5 hours, resulting in a five-fold speed improvement. The prediction performance of the prioritized sampling algorithm remains comparable with that of the naive MCMC: the resulting R^2 and MSE from the two algorithms were almost identical, with a correlation above 0.995 across 20 data splitting replicates. Note that the prioritizing sampling strategy we employ in DPR differs from the sample strategy used in BayesR[3], where a different set of M SNPs are used every T iteration. Indeed, our sampling strategy is still guaranteed to reach the same stationary distribution given a large number of iterations, regardless which set of M SNPs or which set of M and T values we choose to perform prioritized sampling.

Finally, we follow the truncated stick-breaking approximation approach of Blei and Jordan[12,13] and approximate the infinite normal mixture by a truncated normal mixture with K normal components. To ensure that π_k is well defined under the truncated

approximation (i.e. $\sum_{k=1}^{K}\pi_k = 1$), we set $v_k = 1$, $1 - v_k = 0$ for $k>K$[12,14,15]. With the truncated Dirichlet process approximation, we can draw posteriors via a simple Gibbs sampler, thus alleviating much of the computational burden associated with sampling the full Dirichlet process conditionally through the Chinese restaurant process. Because different truncated normal mixture approximations may result in different accuracy, we treat K as a parameter and use the deviance information criterion (DIC)[15-17] to select the optimal K automatically. To do so, we first perform MCMC sampling on a grid of K values from 2 to 10. For each K, we compute DIC using a small number of MCMC iterations (5,000). We select the optimal DPR model with the smallest DIC. We then run a large number of MCMC iterations (50,000) with the optimal DPR model. This strategy makes the selection of K in our DPR adaptive, while keeping computational cost in check. Note that this selection strategy may lead to local optimal and consequently hinders the performance of our method. Alternative and better strategies may improve DPR's prediction performance further. For the final 50,000 MCMC iterations, we discarded the first 10,000 as burn in and kept the remaining 40,000 for parameter estimation. We did not thin the MCMC chain[18], which may help improve prediction performance further. Finally, we also provided trace-plots for the log posterior likelihood of our model in all real data analyses following the recommendation in[15,19]. These trace-plots serve as a summary assessment of parameter convergence.

Mean Field Variational Inference for DPR

Despite the many algorithm innovations we use, the resulting MCMC algorithm is still computationally heavy. Therefore, we develop an alternative, much faster, algorithm based on variational Bayesian approximation[12,20-23]. Variational Bayesian approximation attempts to approximate the joint posterior distribution by a variational distribution, $q(\boldsymbol{\theta}) = \prod_j q(\theta_j)$, that assumes posterior independence among parameters θ_j. To do so, we minimize the Kullback-Leibler (KL) divergence between $p(\boldsymbol{\theta}|\mathbf{y})$ and $q(\boldsymbol{\theta})$

$$\begin{aligned}\mathrm{KL}(q(\boldsymbol{\theta})\,|\,p(\boldsymbol{\theta}|\mathbf{y})) &= E_{q(\boldsymbol{\theta})}\left(\log\frac{q(\boldsymbol{\theta})}{p(\boldsymbol{\theta}|\mathbf{y})}\right), \\ &= E_{q(\boldsymbol{\theta})}(\log q(\boldsymbol{\theta})) - E_{q(\boldsymbol{\theta})}(\log p(\boldsymbol{\theta},\mathbf{y})) + \log p(\mathbf{y}).\end{aligned} \quad (27)$$

Because the marginal probability $\log p(\mathbf{y})$ does not depend on the variational distribution, minimizing the KL divergence is equivalent to maximizing the evidence lower bound (ELBO)

$$E_{q(\boldsymbol{\theta})}(\log p(\boldsymbol{\theta},\mathbf{y})) - E_{q(\boldsymbol{\theta})}(\log q(\boldsymbol{\theta})). \tag{28}$$

To obtain the variational approximation, we can use the gradient ascent algorithm to maximize the above quantity with respect to each θ_j in turn. For each θ_j, we set the following derivative

$$\begin{aligned}&\frac{\partial E_{q(\boldsymbol{\theta})}(\log p(\boldsymbol{\theta},\mathbf{y})) - E_{q(\boldsymbol{\theta})}(\log q(\boldsymbol{\theta}))}{\partial q(\theta_j)}\\ &\quad= \frac{\partial\left(\int q(\theta_j) E_{q(-\theta_j)}(\log p(\boldsymbol{\theta},\mathbf{y}))d\theta_j - \int q(\theta_j)\log q(\theta_j)d\theta_j\right)}{\partial q(\theta_j)}\\ &\quad= E_{q(-\theta_j)}(\log p(\boldsymbol{\theta},\mathbf{y})) - \log q(\theta_j) - 1\end{aligned} \tag{29}$$

to zero. Because $p(\boldsymbol{\theta},\mathbf{y})$ does not contain any parameter in $q(\theta_j)$, this leads to an update for each θ_j in the following form

$$q(\theta_j) \propto e^{E_{q(-\theta_j)}(\log p(\theta,\mathbf{y}))} \propto e^{E_{q(-\theta_j)}(\log p(\theta_j|\theta_{-j},\mathbf{y}))}. \tag{30}$$

Inference based on the above factorized form of the variational distribution is commonly known as the mean field variational Bayesian approximation inference[20,21,23-25].

We apply the mean field variational Bayesian approximation to DPR. Because computing ELBO is difficult for non-analytic variational distributions[26,27], we cannot integrate out **u** from model (5) as we do for MCMC. Instead, we keep **u**. We also denote $\mathbf{g} = \mathbf{U}^T\mathbf{u}$. Our joint log posterior is

$$
\begin{aligned}
\log p(\boldsymbol{\theta},\mathbf{y}) &= \log p(\mathbf{y}\,|\,\boldsymbol{\alpha},\boldsymbol{\beta},\mathbf{u}) + \log p(\boldsymbol{\beta}\,|\,\boldsymbol{\gamma},\sigma_k^2,\sigma_e^2) + \log p(\mathbf{u}\,|\,\sigma_b^2,\sigma_e^2) \\
&+ \log p(\boldsymbol{\gamma}\,|\,\nu_k) + \log p(\nu_k\,|\,\lambda) + \log p(\sigma_k^2\,|\,a_{0k},b_{0k}) + \log p(\sigma_e^2\,|\,a_{0e},b_{0e}) \\
&+ \log p(\sigma_b^2\,|\,a_{0b},b_{0b}) + \log p(\lambda\,|\,a_{0\lambda},b_{0\lambda}) \\
&= C - \frac{n}{2}\log(\sigma_e^2) - \frac{1}{2\sigma_e^2}(\mathbf{y}-\mathbf{W}\boldsymbol{\alpha}-\mathbf{X}\boldsymbol{\beta}-\mathbf{u})^T(\mathbf{y}-\mathbf{W}\boldsymbol{\alpha}-\mathbf{X}\boldsymbol{\beta}-\mathbf{u}) \\
&+ \sum_i\sum_{k=2}^{\infty}\gamma_{ik}\left(-\frac{1}{2}\log(\sigma_e^2) - \frac{1}{2}\log(\sigma_k^2) - \frac{\beta_{ik}^2}{2\sigma_k^2\sigma_e^2}\right) \\
&- \frac{n}{2}\log(\sigma_e^2) - \frac{n}{2}\log(\sigma_b^2) - \frac{1}{2}\log|\mathbf{K}| - \frac{1}{2}\mathbf{u}^T(\sigma_e^2\sigma_b^2\mathbf{K})^{-1}\mathbf{u} \\
&+ \sum_i\sum_{k=1}^{\infty}\gamma_{ik}\left(\log(\nu_k) + \sum_{l=1}^{k-1}\log(1-\nu_l)\right) + \sum_k^{\infty}\left((\lambda-1)\log(1-\nu_k) + \log(\lambda)\right) \\
&- \sum_k^{\infty}(a_{0k}+1)\log(\sigma_k^2) - \sum_k^{\infty}b_{0k}\sigma_k^{-2} - (a_{0e}+1)\log(\sigma_e^2) - b_{0e}\sigma_e^{-2} \\
&- (a_{0b}+1)\log(\sigma_b^2) - b_{0b}\sigma_b^{-2} + (a_{0\lambda}-1)\log(\lambda) - b_{0\lambda}\lambda,
\end{aligned} \tag{31}
$$

where again C is a normalizing constant. We will ignore the constant terms in the following updates.

We follow the truncated stick-breaking approximation approach of Blei and Jordan[12] and use a finite mixture with a fixed number of normal components, K, as an approximation to the posterior distribution. The parameter K here is considered as a variational parameter and we choose K by optimizing ELBO. Note again that although we use a finite mixture as an approximation to the posterior distribution, our likelihood still consists of a mixture of infinitely many normal distributions[12]. To choose K, we perform variational inference with DPR on different K values ranging from 2 to 10. Following[12], we then choose the optimal DPR model with the largest ELBO and we present results based on the optimal DPR.

Variational distribution for α_j

First, for α_j, we have

$$
\begin{aligned}
\log q(\alpha_j) &= -\frac{E(\sigma_e^{-2})\mathbf{w}_j{}^T\mathbf{w}_j}{2}\alpha_j^2 \\
&+ E(\sigma_e^{-2})\mathbf{w}_j{}^T\left(\mathbf{y} - \sum_{m\neq j}\mathbf{w}_m E(\alpha_m) - \mathbf{X}E(\boldsymbol{\beta}) - E(\mathbf{u})\right)\alpha_j.
\end{aligned} \tag{32}
$$

Therefore, the variation distribution for α_j is $q(\alpha_j) = N(m_j, s_j^2)$, where

$$m_j = \frac{\mathbf{w}_j^T(\mathbf{y} - \sum_{m \neq j} \mathbf{w}_m E(\alpha_m) - \mathbf{X}E(\boldsymbol{\beta}) - E(\mathbf{u}))}{\mathbf{w}_j^T \mathbf{w}_j},$$
$$s_j^2 = \frac{E(\sigma_e^{-2})^{-1}}{\mathbf{w}_j^T \mathbf{w}_j}. \quad (33)$$

Variational distributions for β_{ik} and γ_{ik}

For β_{ik} and γ_{ik}, we have

$$\begin{aligned}\log q(\beta_{ik}, \gamma_{ik}) = &-\frac{E(\sigma_e^{-2})\mathbf{x}_i^T \mathbf{x}_i}{2} E(\beta_i^2) \\ &+ E(\sigma_e^{-2})\mathbf{x}_i^T(\mathbf{y} - \mathbf{W}E(\boldsymbol{\alpha}) - \sum_{m \neq i} \mathbf{x}_m E(\beta_m) - E(\mathbf{u}))\beta_i \\ &+ \gamma_{ik}(-\frac{1}{2}\log E(\sigma_e^2) - \frac{1}{2}\log E(\sigma_k^2) - \frac{1}{2}E(\sigma_k^{-2})E(\sigma_e^{-2})\beta_{ik}^2) \\ &+ \gamma_{ik}(\log E(\nu_k) + \sum_{l=1}^{k-1} \log E(1-\nu_l)).\end{aligned} \quad (34)$$

A natural update form for $q(\beta_{ik}, \gamma_{ik})$ is thus

$$q(\beta_{ik} \mid \gamma_{ik} = 1) = N(m_{ik}, s_{ik}^2),$$
$$q(\gamma_{ik} = 1) = \varphi_{ik} \propto e^{m_{ik}^2/2s_{ik}^2 + \log(s_{ik}) - E(\log(\sigma_e)) - E(\log(\sigma_k)) + E(\log(\nu_k)) + \sum_{l=1}^{k-1} E(\log(1-\nu_l))}, \quad (35)$$

where

$$m_{ik} = \frac{\mathbf{x}_i^T(\mathbf{y} - \mathbf{W}E(\boldsymbol{\alpha}) - \sum_{m \neq i} \mathbf{x}_m E(\beta_m) - E(\mathbf{u}))}{\mathbf{x}_i^T \mathbf{x}_i + E(\sigma_k^{-2})},$$
$$s_{ik}^2 = \frac{E(\sigma_e^{-2})^{-1}}{\mathbf{x}_i^T \mathbf{x}_i + E(\sigma_k^{-2})}. \quad (36)$$

Variational distribution for v

For *v,* we have

$$\log q(\nu_k) = \sum_i E(\gamma_{ik}) \log(\nu_k) + \sum_i \sum_{l=k+1}^{\infty} E(\gamma_{il}) \log(1-\nu_k) + (E(\lambda) - 1)\log(1-\nu_k). \quad (37)$$

Thus $q(\nu_k) = \mathrm{Beta}(\kappa_k, \lambda_k)$, where

$$\kappa_k = \sum_i E(\gamma_{ik}) + 1,$$
$$\lambda_k = \sum_i \sum_{l=k+1}^{\infty} E(\gamma_{il}) + E(\lambda). \quad (38)$$

Variational distribution for σ_k^2

For σ_k^2, we have

$$\log q(\sigma_k^2) = -\left(\frac{\sum_i E(\gamma_{ik})}{2} + a_{0k} + 1\right)\log(\sigma_k^2) - \left(\frac{\sum_i E(\gamma_{ik}\beta_{ik}^2)E(\sigma_e^{-2})}{2} + b_{0k}\right)\sigma_k^{-2}. \quad (39)$$

Thus $q(\sigma_k^2) = \text{inverse-gamma}(a_k, b_k)$, where

$$a_k = \frac{1}{2}\sum_i E(\gamma_{ik}) + a_{0k},$$
$$b_k = \frac{1}{2}\sum_i E(\gamma_{ik}\beta_{ik}^2)E(\sigma_e^{-2}) + b_{0k}. \quad (40)$$

Variational distribution for λ

For λ, we have

$$\log q(\lambda) = \lambda\left(\sum_k^{\infty} \log E(1-\nu_k) - b_{0\lambda}\right) + \log(\lambda)\left(a_{0\lambda} + \sum_k^{\infty} 1_k\right). \quad (41)$$

Thus $q(\lambda) = \text{gamma}(a_\lambda, b_\lambda)$, where

$$a_\lambda = a_{0\lambda} + \sum_k^{\infty} 1_k,$$
$$b_\lambda = b_{0\lambda} - \sum_k^{\infty} \log E(1-\nu_k). \quad (42)$$

Variational distribution for g

For **g**, we have

$$\log q(\mathbf{g}) = -\frac{1}{2\sigma_e^2}(\mathbf{U}^T\mathbf{y} - \mathbf{U}^T\mathbf{W}E(\boldsymbol{\alpha}) - \mathbf{U}^T\mathbf{X}E(\boldsymbol{\beta}) - \mathbf{g})^T(\mathbf{U}^T\mathbf{y} - \mathbf{U}^T\mathbf{W}E(\boldsymbol{\alpha}) - \mathbf{U}^T\mathbf{X}E(\boldsymbol{\beta}) - \mathbf{g})$$
$$-\frac{1}{2}\mathbf{g}^T(\sigma_e^2\sigma_b^2\mathbf{D})^{-1}\mathbf{g}. \quad (43)$$

Thus $q(\mathbf{g}) = \text{MVN}_n(\boldsymbol{\mu}, \boldsymbol{\Sigma})$, where

$$\begin{aligned}\boldsymbol{\mu} &= (E(\sigma_b^{-2}\mathbf{D}^{-1}) + \mathbf{I}_n)^{-1}(\mathbf{U}^T\mathbf{y} - \mathbf{U}^T\mathbf{W}E(\boldsymbol{\alpha}) - \mathbf{U}^T\mathbf{X}E(\boldsymbol{\beta})),\\ \boldsymbol{\Sigma} &= (E(\sigma_b^{-2}\mathbf{D}^{-1}) + \mathbf{I}_n)^{-1}E(\sigma_e^{-2})^{-1}.\end{aligned} \tag{44}$$

Here, the covariance matrix is diagonal, which facilitates computation. As in MCMC, we use the relationship in equation (4) to obtain the mean of $\mathbf{b}$ at the end of the algorithm. The estimated mean of $\mathbf{b}$ is added back to the mean of $\boldsymbol{\beta}$ to obtain a mean estimate for $\tilde{\boldsymbol{\beta}}$.

Variational distribution for σ_b^2

For σ_b^2, we have

$$\log q(\sigma_b^2) = -\frac{n}{2}\log(\sigma_b^2) - \frac{1}{2}\sum_i \sigma_b^{-2}E(g_i)^2 / E(d_i\sigma_e^2) - (a_{0b}+1)\log(\sigma_b^2) - b_{0b}\sigma_b^{-2}, \tag{45}$$

where d_i is the ith diagonal element of $\mathbf{D}$. Thus $q(\sigma_b^2) = \text{inverse-gamma}(a_b, b_b)$, where

$$\begin{aligned}a_b &= \frac{n}{2} + a_{0b},\\ b_b &= \frac{1}{2}\sum_i E(g_i)^2 E(d_i^{-1}\sigma_e^{-2}) + b_{0b}.\end{aligned} \tag{46}$$

Variational distribution for σ_e^2

Finally, for σ_e^2, we have

$$\begin{aligned}\log q(\sigma_e^2) = &-(n + \sum_i\sum_{k=2} E(\gamma_{ik})/2 + a_{0e} + 1)\log(\sigma_e^2) - \frac{1}{2}A \times \sigma_e^{-2}\\ &-\frac{1}{2}(\sum_i\sum_k E(\gamma_{ik}\beta_{ik}^2)E(\sigma_k^{-2}) + \sum_i E(g_i)^2 / E(d_i\sigma_b^2) + 2b_{0e})\sigma_e^{-2}.\end{aligned} \tag{47}$$

Thus $q(\sigma_e^2) = \text{inverse-gamma}(a_e, b_e)$, where

$$\begin{aligned}a_e &= n + \sum_i\sum_{k=2} E(\gamma_{ik})/2 + a_{0e},\\ b_e &= \frac{1}{2}(A + \sum_i\sum_{k=2} E(\gamma_{ik}\beta_{ik}^2)E(\sigma_k^{-2}) + \sum_i E(g_i)^2 E(\sigma_b^{-2}d_i^{-1}) + 2b_{0e}),\\ A &= (\mathbf{U}^T\mathbf{y} - \mathbf{U}^T\mathbf{W}E(\boldsymbol{\alpha}) - \mathbf{U}^T\mathbf{X}E(\boldsymbol{\beta}) - E(\mathbf{g}))^T(\mathbf{U}^T\mathbf{y} - \mathbf{U}^T\mathbf{W}E(\boldsymbol{\alpha}) - \mathbf{U}^T\mathbf{X}E(\boldsymbol{\beta}) - E(\mathbf{g}))\\ &\quad + \sum_j \mathbf{w}_j^T\mathbf{w}_j s_j^2 + \sum_i \boldsymbol{\Sigma}_{ii} + \sum_i \mathbf{x}_i^T\mathbf{x}_i(\sum_k E(\gamma_{ik})(m_{ik}^2 + s_{ik}^2) - (\sum_k E(\gamma_{ik})m_{ik})^2),\end{aligned} \tag{48}$$

where $\boldsymbol{\Sigma}_{ii}$ is the ith diagonal element of $\boldsymbol{\Sigma}$ given in (44).

To evaluate all the above expectations, we need

$$
\begin{aligned}
E_{q(\nu_k)}(\log(\nu_k)) &= \Psi(\kappa_k) - \Psi(\kappa_k + \lambda_k), \\
E_{q(\nu_k)}(\log(1-\nu_k)) &= \Psi(\lambda_k) - \Psi(\kappa_k + \lambda_k), \\
E_{q(\gamma_i, \tilde{\beta}_i)}(\gamma_i \beta_i^2) &= \sum_k \varphi_{ik}(m_{ik}^2 + s_{ik}^2), \\
E_{q(\gamma_i, \tilde{\beta}_i)}(\beta_i) &= \sum_k \varphi_{ik} m_{ik}, \\
E_{q(\alpha_j)}(\alpha_j^2) &= m_j^2 + s_j^2, \\
E_{q(\alpha_j)}(\alpha_j) &= m_j, \\
E(\mathbf{g}) &= \boldsymbol{\mu}, \\
E_{q(\sigma_k^2)}(\log \sigma_k) &= \frac{1}{2}(\log(b_k) - \Psi(a_k)), \\
E_{q(\sigma_k^2)}(\sigma_k^{-2}) &= \frac{a_k}{b_k}, \\
E_{q(\lambda)}(\log \lambda) &= \Psi(a_\lambda) - \log(b_\lambda), \\
E_{q(\lambda)}(\lambda) &= a_\lambda / b_\lambda,
\end{aligned}
\tag{49}
$$

where Ψ is the digamma function.

ELBO and convergence

We use ELBO to check convergence of the variational algorithm. For the explicit form of ELBO, first, we have

$$
\begin{aligned}
E_{q(\beta_i,\gamma_i)}(\log(q(\beta_i,\gamma_i))) &= \sum_{k=2} \varphi_{ik}(\log \varphi_{ik} - \frac{1}{2}\log(2\pi \times e \times s_{ik}^2) - \frac{1}{2}), \\
E_{q(\alpha_j)}(\log(q(\alpha_j))) &= -\frac{1}{2}\log(s_j^2), \\
E_{q(g_i)}(\log(q(g_i))) &= -\frac{1}{2}\log(\boldsymbol{\Sigma}_{ii}), \\
E_{q(\nu_k)}(\log(q(\nu_k))) &= \log\Gamma(\kappa_k + \lambda_k) - \log\Gamma(\kappa_k) - \log\Gamma(\lambda_k) \\
&\quad + (\kappa_k - 1)(\Psi(\kappa_k) - \Psi(\kappa_k + \lambda_k)) \\
&\quad + (\lambda_k - 1)(\Psi(\lambda_k) - \Psi(\kappa_k + \lambda_k)), \\
E_{q(\sigma_k^2)}(\log(q(\sigma_k^2))) &= a_k \log b_k - \log\Gamma(a_k) + (a_k + 1)(\Psi(a_k) - \log b_k) - a_k, \\
E_{q(\sigma_e^2)}(\log(q(\sigma_e^2))) &= a_e \log b_e - \log\Gamma(a_e) + (a_e + 1)(\Psi(a_e) - \log b_e) - a_e, \\
E_{q(\sigma_b^2)}(\log(q(\sigma_b^2))) &= a_b \log b_b - \log\Gamma(a_b) + (a_b + 1)(\Psi(a_b) - \log b_b) - a_b, \\
E_{q(\lambda)}(\log(q(\lambda))) &= \log b_\lambda - \log\Gamma(a_\lambda) - (1 - a_\lambda)\Psi(a_\lambda) - a_\lambda.
\end{aligned}
\tag{50}
$$

In addition, we have

$$\begin{aligned}E_{q(\boldsymbol{\theta})}(\log p(\boldsymbol{\theta},\mathbf{y})) = &-(a_e+1)(\log b_e-\Psi(a_e))-\frac{1}{2}\sum_i\sum_{k=2}\varphi_{ik}(\log b_k-\Psi(a_k))\\ &-(a_{0k}+1)\sum_{k=2}(\log b_k-\Psi(a_k))-(a_b+1)(\log b_b-\Psi(a_b))\\ &-\frac{1}{2}\frac{a_e}{b_e}(A+\sum_i\sum_{k=2}\varphi_{ik}\frac{a_k}{b_k}(m_{ik}^2+s_{ik}^2)+\frac{a_b}{b_b}\sum_i(\mu_i^2+\boldsymbol{\Sigma}_{ii})/d_i+2b_{0e})\\ &+\sum_i\sum_{k=1}\varphi_{ik}(\Psi(\kappa_k)-\Psi(\kappa_k+\lambda_k)+\sum_{l=1}^{k-1}(\Psi(\lambda_l)-\Psi(\kappa_l+\lambda_l)))\\ &+(\frac{a_\lambda}{b_\lambda}-1)(\sum_k(\Psi(\lambda_k)-\Psi(\kappa_k+\lambda_k)))+(a_\lambda-1)(\Psi(a_\lambda)-\log b_\lambda)\\ &-b_{0k}\sum_{k=2}\frac{a_k}{b_k}-b_{0b}\frac{a_b}{b_b}-b_{0\lambda}\frac{a_\lambda}{b_\lambda}.\end{aligned} \tag{51}$$

Finally,

$$\begin{aligned}E_{q(\boldsymbol{\theta})}(\log(q(\boldsymbol{\theta})) = &-(a_e+1)(\log b_e-\Psi(a_e))-a_e\\ &-\sum_k(a_k+1)(\log b_k-\Psi(a_k))\\ &-(a_b+1)(\log b_b-\Psi(a_b))\\ &+\sum_i\sum_{k=1}\varphi_{ik}(\Psi(\kappa_k)-\Psi(\kappa_k+\lambda_k)+\sum_{l=1}^{k-1}(\Psi(\lambda_l)-\Psi(\kappa_l+\lambda_l)))\\ &+(\frac{a_\lambda}{b_\lambda}-1)(\sum_k(\Psi(\lambda_k)-\Psi(\kappa_k+\lambda_k)))+(a_\lambda-1)(\Psi(a_\lambda)-\log b_\lambda).\end{aligned} \tag{52}$$

Therefore, the ELBO is

$$\begin{aligned}\mathrm{ELBO} &= E_{q(\boldsymbol{\theta})}(\log p(\boldsymbol{\theta},\mathbf{y}))-E_{q(\boldsymbol{\theta})}(\log(q(\boldsymbol{\theta}))\\ &=\log\Gamma(a_e)-a_e\log b_e\\ &\quad+\log\Gamma(a_b)-a_b\log b_b+a_b\\ &\quad+\sum_{k=2}(\log\Gamma(a_k)-a_k\log b_k+a_k)\\ &\quad+\sum_k(\log\Gamma(k_k)+\log\Gamma(\lambda_k)-\log\Gamma(k_k+\lambda_k))\\ &\quad-\sum_i\sum_{k=2}\varphi_{ik}(\log\varphi_{ik}-\frac{1}{2}\log(2\pi\times e\times s_{ik}^2)-\frac{1}{2})+\frac{1}{2}\sum_j\log(s_j^2)+\frac{1}{2}\sum_i\log(\boldsymbol{\Sigma}_{ii})\\ &\quad+\log\Gamma(a_\lambda)-a_\lambda\log b_\lambda+a_\lambda-b_{0k}\sum_{k=2}\frac{a_k}{b_k}-b_{0b}\frac{a_b}{b_b}-b_{0\lambda}\frac{a_\lambda}{b_\lambda}.\end{aligned} \tag{53}$$

Supplementary Figures and Tables

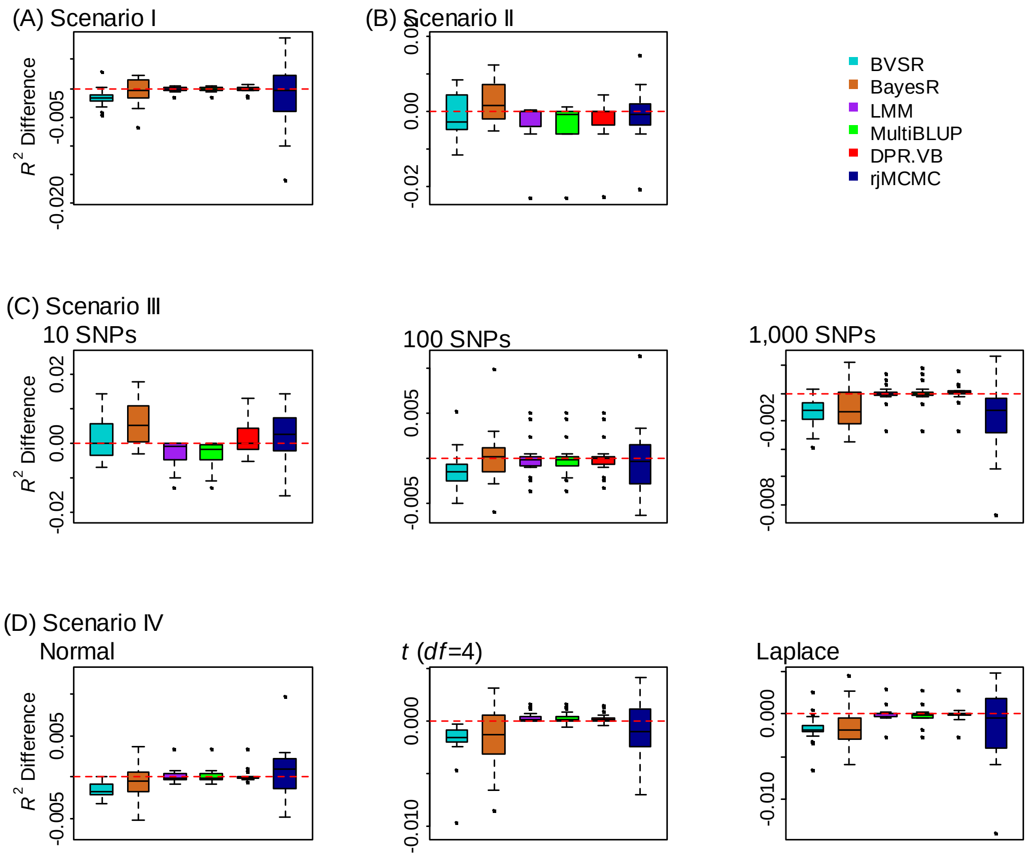

Supplementary Figure 1. Comparison of prediction performance of several methods with DPR.MCMC in simulations when PVE=0.2. Performance is measured by R^2 difference with respect to DPR.MCMC, where a negative value (i.e. values below the red horizontal line) indicates worse performance than DPR.MCMC. The sample R^2 differences are obtained from 20 replicates in each scenario. Methods for comparison include BVSR (cyan), BayesR (chocolate), LMM (purple), MultiBLUP (green), DPR.VB (red), rjMCMC (black blue) and DPR.MCMC. Simulation scenarios include: (A) Scenario I, which satisfies the DPR modeling assumption; (B) Scenario II, which satisfies the BayesR modeling assumption; (C) Scenario III, where the number of SNPs in the large effect group is 10, 100, or 1,000; and (D) Scenario IV, where the effect sizes are generated from either a normal distribution, a t-distribution or a Laplace distribution. For each box plot, the bottom and top of the box are the first and third quartiles, while the

ends of whiskers represent either the lowest datum within 1.5 interquartile range of the lower quartile or the highest datum within 1.5 interquartile range of the upper quartile. For DPR.MCMC, the mean predictive R^2 in the test set and the standard deviation for the eight settings are respectively 0.074 (0.020), 0.081 (0.016), 0.076 (0.018), 0.072 (0.019), 0.064 (0.016), 0.083 (0.023), 0.077 (0.016) and 0.077 (0.017).

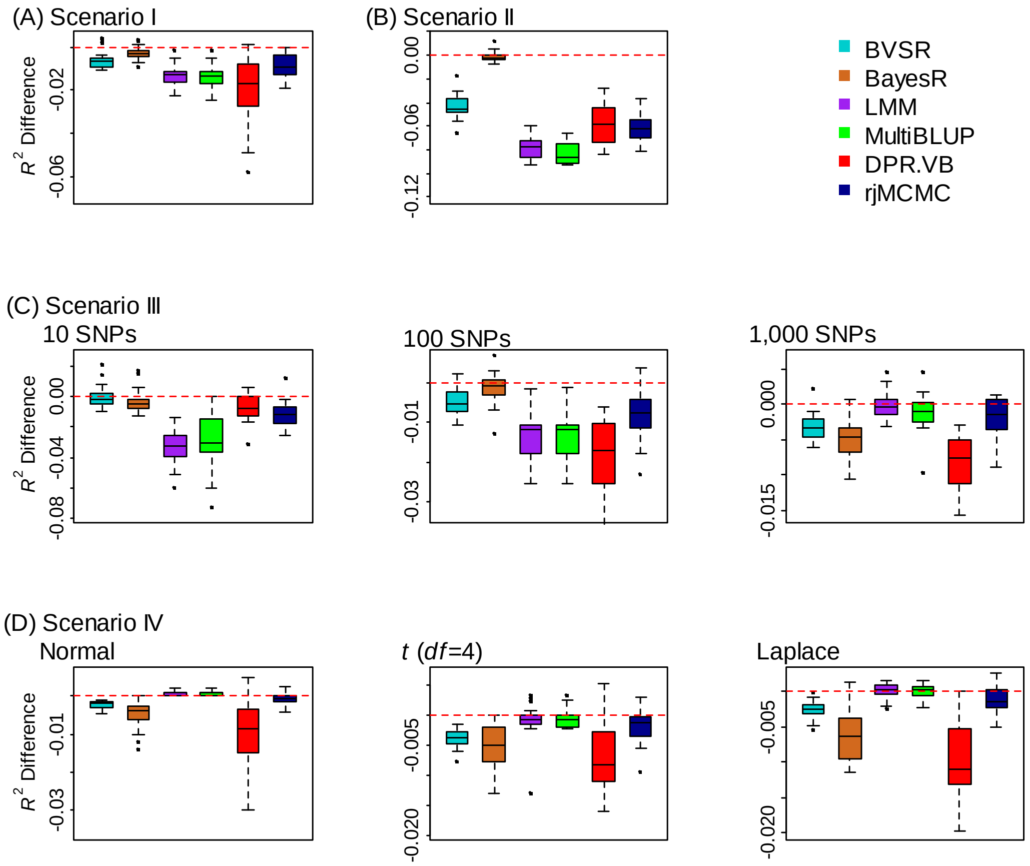

Supplementary Figure 2. Comparison of prediction performance of several methods with DPR.MCMC in simulations when PVE=0.8. Performance is measured by R^2 difference with respect to DPR.MCMC, where a negative value (i.e. values below the red horizontal line) indicates worse performance than DPR.MCMC. The sample R^2 differences are obtained from 20 replicates in each scenario. Methods for comparison include BVSR (cyan), BayesR (chocolate), LMM (purple), MultiBLUP (green), DPR.VB (red), rjMCMC (black blue) and DPR.MCMC. Simulation scenarios include: (A) Scenario I, which satisfies the DPR modeling assumption; (B) Scenario II, which satisfies the BayesR modeling assumption; (C) Scenario III, where the number of SNPs in the large effect group is 10, 100, or 1,000; and (D) Scenario IV, where the effect sizes are generated from either a normal distribution, a t-distribution or a Laplace distribution. For each box plot, the bottom and top of the box are the first and third quartiles, while the ends of whiskers represent either the lowest datum within 1.5 interquartile range of the lower quartile or the highest datum within 1.5 interquartile range of the upper quartile.

For DPR.MCMC, the mean predictive R^2 in the test set and the standard deviation for the eight settings are respectively 0.554 (0.028), 0.622 (0.022), 0.569 (0.023), 0.548 (0.027), 0.537 (0.030), 0.543 (0.028), 0.546 (0.027) and 0.539 (0.022).

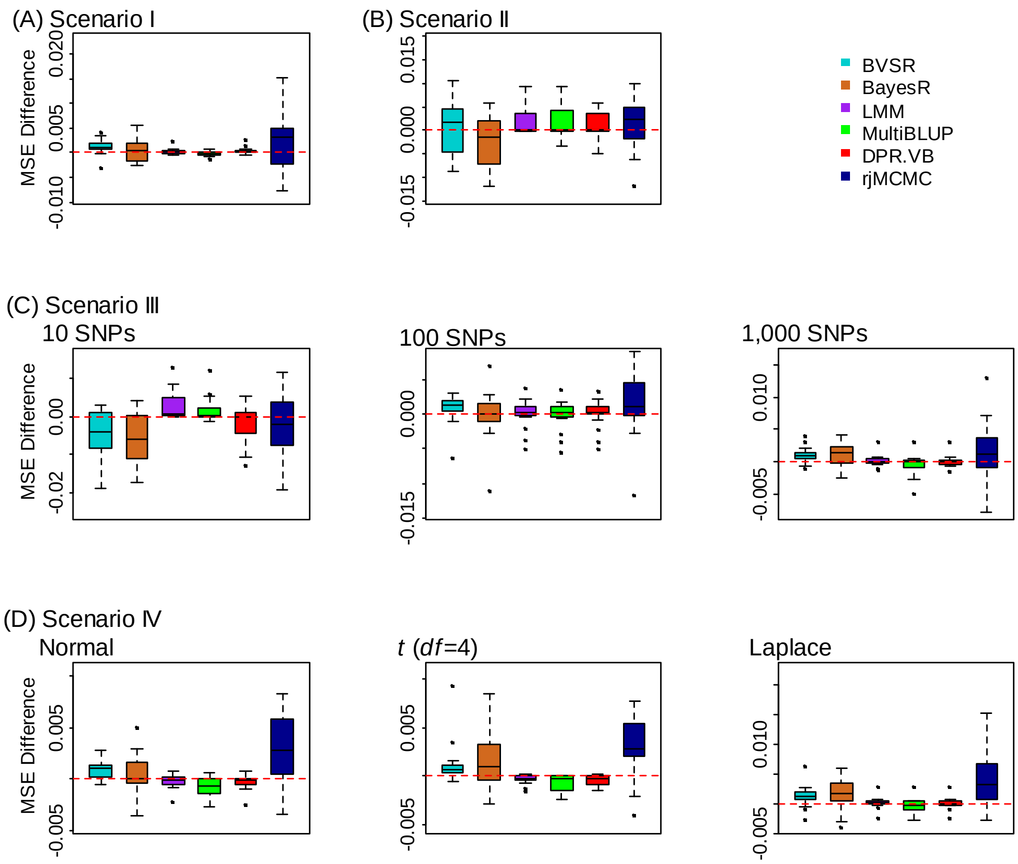

Supplementary Figure 3. Comparison of prediction performance of several methods with DPR.MCMC in simulations when PVE=0.2. Performance is measured by MSE difference with respect to DPR.MCMC, where a positive value (i.e. values above the red horizontal line) indicates worse performance than DPR.MCMC. The sample MSE differences are obtained from 20 replicates in each scenario. Methods for comparison include BVSR (cyan), BayesR (chocolate), LMM (purple), MultiBLUP (green), DPR.VB (red), rjMCMC (black blue) and DPR.MCMC. Simulation scenarios include: (A) Scenario I, which satisfies the DPR modeling assumption; (B) Scenario II, which satisfies the BayesR modeling assumption; (C) Scenario III, where the number of SNPs in the large effect group is 10, 100, or 1,000; and (D) Scenario IV, where the effect sizes are generated from either a normal distribution, a t-distribution or a Laplace distribution. For each box plot, the bottom and top of the box are the first and third quartiles, while the ends of whiskers represent either the lowest datum within 1.5 interquartile range of the lower quartile or the highest datum within 1.5 interquartile range of the upper quartile.

For DPR.MCMC, the mean predictive MSE in the test set and the standard deviation for the eight settings are respectively 0.919 (0.044), 0.910 (0.038), 0.929 (0.036), 0.944 (0.053), 0.923 (0.038), 0.925 (0.033), 0.924 (0.037) and 0.918 (0.037).

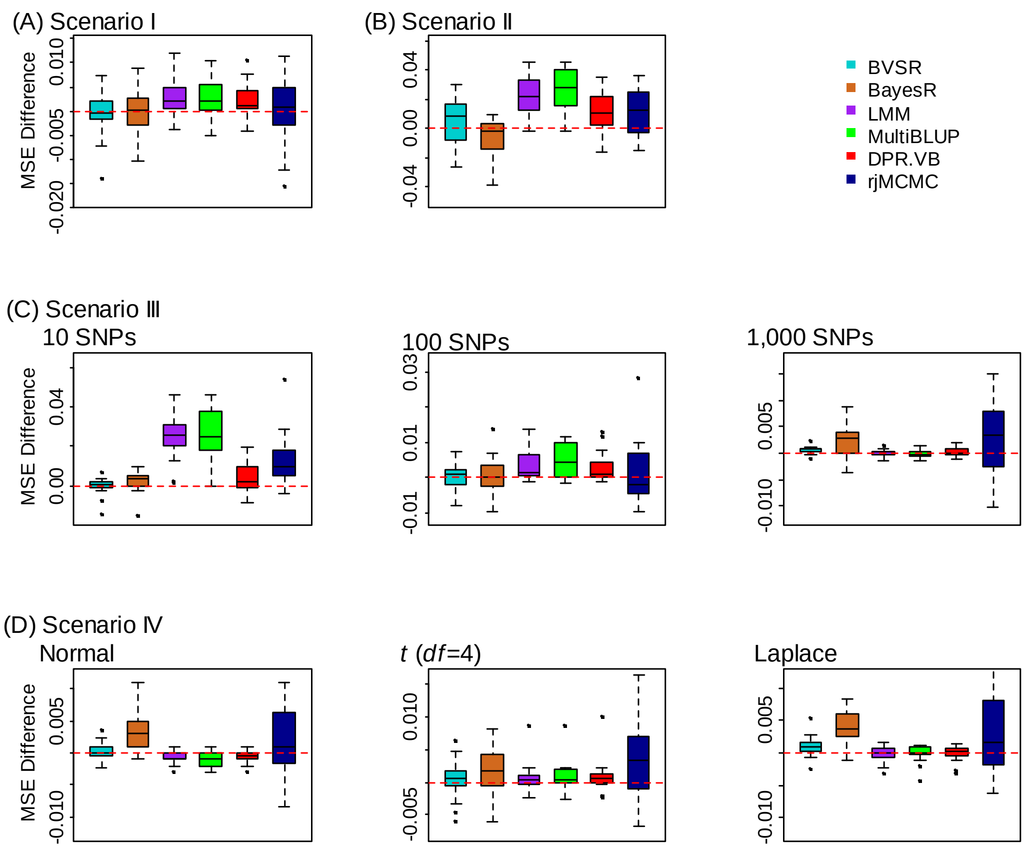

Supplementary Figure 4. Comparison of prediction performance of several methods with DPR.MCMC in simulations when PVE=0.5. Performance is measured by MSE difference with respect to DPR.MCMC, where a positive value (i.e. values above the red horizontal line) indicates worse performance than DPR.MCMC. The sample MSE differences are obtained from 20 replicates in each scenario. Methods for comparison include BVSR (cyan), BayesR (chocolate), LMM (purple), MultiBLUP (green), DPR.VB (red), rjMCMC (black blue) and DPR.MCMC. Simulation scenarios include: (A) Scenario I, which satisfies the DPR modeling assumption; (B) Scenario II, which satisfies the BayesR modeling assumption; (C) Scenario III, where the number of SNPs in the large effect group is 10, 100, or 1,000; and (D) Scenario IV, where the effect sizes are generated from either a normal distribution, a t-distribution or a Laplace distribution. For each box plot, the bottom and top of the box are the first and third quartiles, while the ends of whiskers represent either the lowest datum within 1.5 interquartile range of the lower quartile or the highest datum within 1.5 interquartile range of the upper quartile.

For DPR.MCMC, the mean predictive MSE in the test set and the standard deviation for the eight settings are respectively 0.722 (0.043), 0.701 (0.028), 0.707 (0.034), 0.717 (0.037), 0.727 (0.034), 0.734 (0.040), 0.721 (0.032) and 0.720 (0.028).

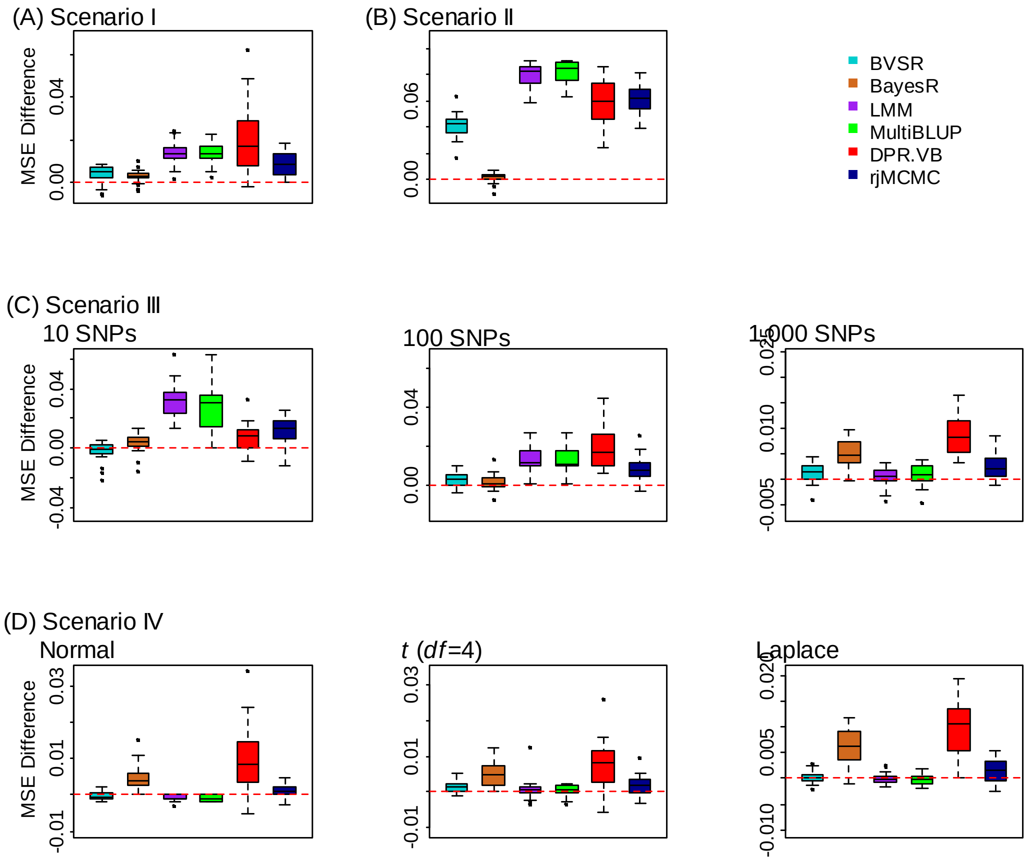

Supplementary Figure 5. Comparison of prediction performance of several methods with DPR.MCMC in simulations when PVE=0.8. Performance is measured by MSE difference with respect to DPR.MCMC, where a positive value (i.e. values above the red horizontal line) indicates worse performance than DPR.MCMC. The sample MSE differences are obtained from 20 replicates in each scenario. Methods for comparison include BVSR (cyan), BayesR (chocolate), LMM (purple), MultiBLUP (green), DPR.VB (red), rjMCMC (black blue) and DPR.MCMC. Simulation scenarios include: (A) Scenario I, which satisfies the DPR modeling assumption; (B) Scenario II, which satisfies the BayesR modeling assumption; (C) Scenario III, where the number of SNPs in the large effect group is 10, 100, or 1,000; and (D) Scenario IV, where the effect sizes are generated from either a normal distribution, a t-distribution or a Laplace distribution. For each box plot, the bottom and top of the box are the first and third quartiles, while the ends of whiskers represent either the lowest datum within 1.5 interquartile range of the lower quartile or the highest datum within 1.5 interquartile range of the upper quartile.

For DPR.MCMC, the mean predictive MSE in the test set and the standard deviation for the eight settings are respectively 0.443 (0.032), 0.379 (0.016), 0.429 (0.024), 0.454 (0.023), 0.464 (0.030), 0.465 (0.027), 0.454 (0.032) and 0.457 (0.022).

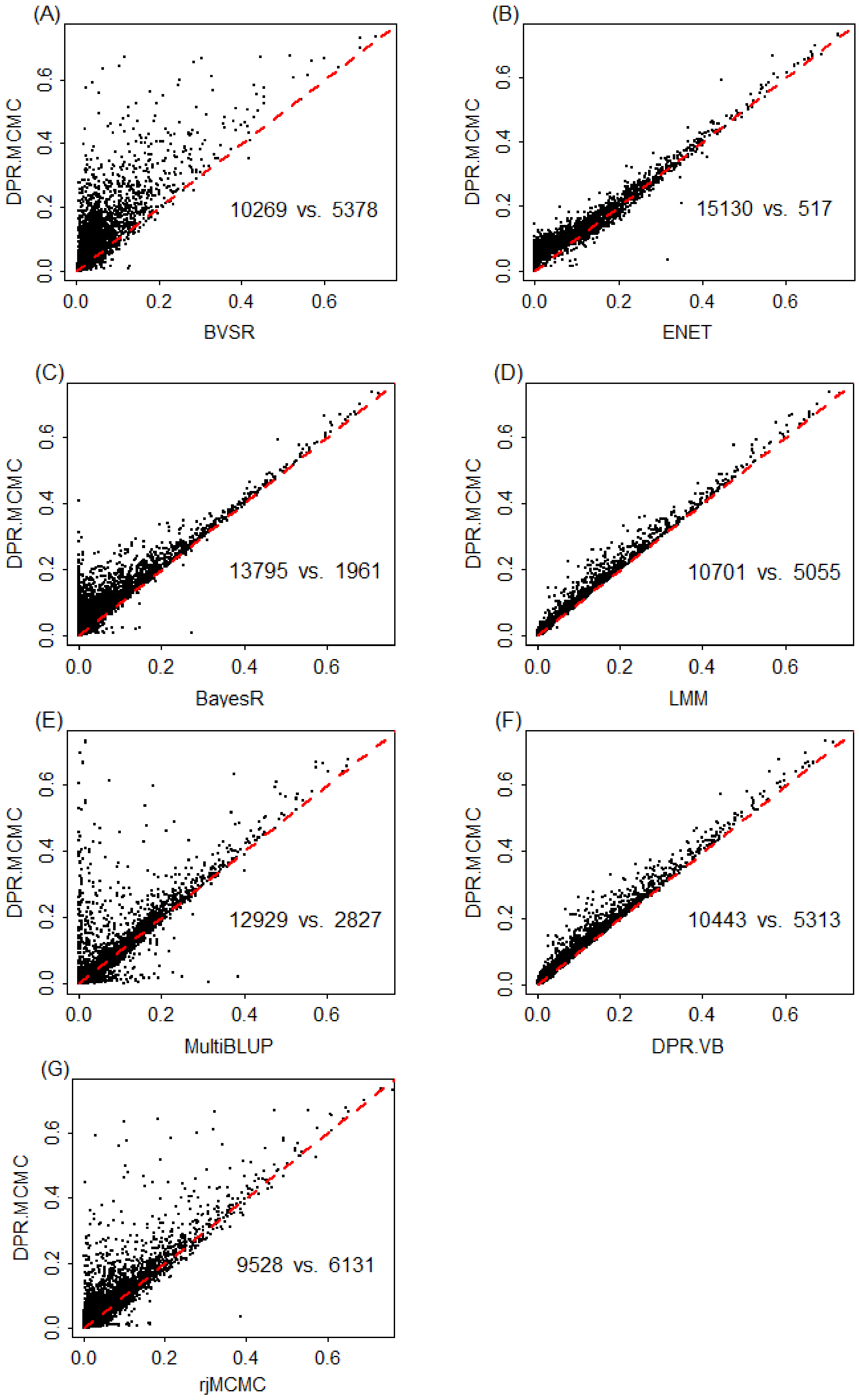

(A)
DPR.MCMC
BVSR
10269 vs. 5378
(B)
DPR.MCMC
ENET
15130 vs. 517
(C)
DPR.MCMC
BayesR
13795 vs. 1961
(D)
DPR.MCMC
LMM
10701 vs. 5055
(E)
DPR.MCMC
MultiBLUP
12929 vs. 2827
(F)
DPR.MCMC
DPR.VB
10443 vs. 5313
(G)
DPR.MCMC
rjMCMC
9528 vs. 6131

Supplementary Figure 6. Comparison of predictive R^2 from DPR.MCMC with the other six methods for predicting gene expression levels in the GEUVADIS data. Scatter plots show (A) predictive R^2 in the test data obtained by DPR.MCMC vs that obtained by BVSR for all genes; (B) DPR.MCMC vs ENET; (C) DPR.MCMC vs BayesR; (D) DPR.MCMC vs LMM; (E) DPR.MCMC vs MultiBLUP; (F) DPR.MCMC vs DPR.VB; (G) DPR.MCMC vs rjMCMC. Each panel also lists the number of genes where DPR.MCMC performs better (first number) and the number of genes where DPR.MCMC performs worse (second number).

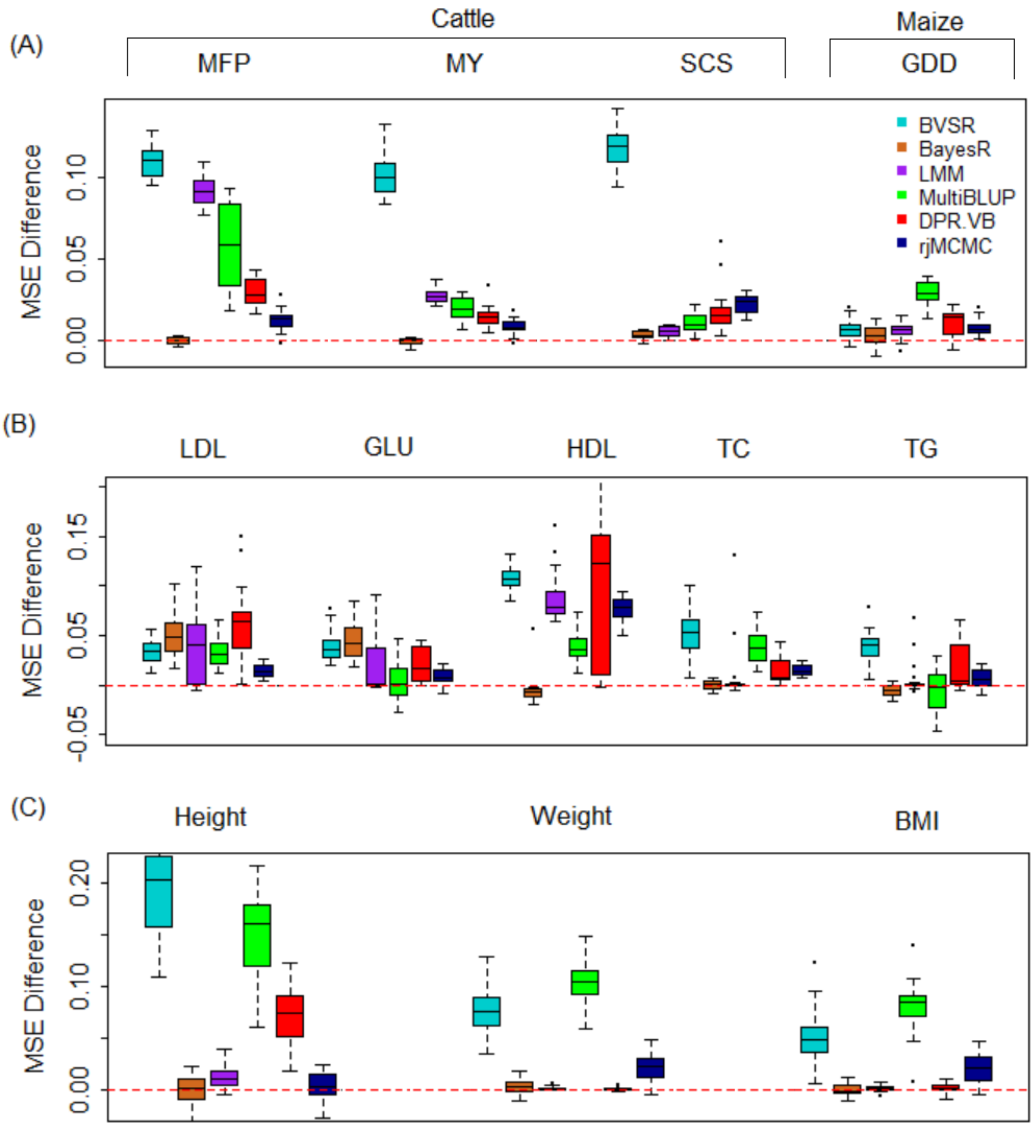

Supplementary Figure 7. Comparison of prediction performance of several methods with DPR.MCMC for twelve traits from three data sets. Performance is measured by MSE difference with respect to DPR.MCMC, where a positive value (i.e. values above the red horizontal line) indicates worse performance than DPR.MCMC. Methods for comparison include BVSR (cyan), BayesR (chocolate), LMM (purple), MultiBLUP (green), DPR.VB (red), rjMCMC (black blue) and DPR.MCMC. The sample MSE differences are obtained from 20 replicates of Monte Carlo cross validation for each trait. For each box plot, the bottom and top of the box are the first and third quartiles, while the ends of whiskers represent either the lowest datum within 1.5 interquartile range of the

lower quartile or the highest datum within 1.5 interquartile range of the upper quartile. For DPR.MCMC, the mean predictive MSE in the test set and the standard deviation are 0.246 (0.011) for MFP, 0.371 (0.019) for MY, 0.446 (0.028) for SCS, 0.170 (0.012) for GDD, 0.928 (0.029) for LDL, 0.954 (0.034) for GLU, 0.833 (0.063) for HDL, 0.970 (0.044) for TC, 0.960 (0.035) for TG, 0.519 (0.050) for height, 0.834 (0.065) for weight and 0.868 (0.074) for BMI. The SNP heritability estimates are 0.912 (0.007) for MFP, 0.810 (0.012) for MY, 0.801 (0.012) for SCS, 0.880 (0.013) for GDD, 0.397 (0.024) for LDL, 0.357 (0.036) for GLU, 0.418 (0.024) for HDL, 0.402 (0.036) for TC, 0.334 (0.034) for TG, 0.905 (0.013) for Height, 0.548 (0.022) for Weight and 0.483 (0.023) for BMI.

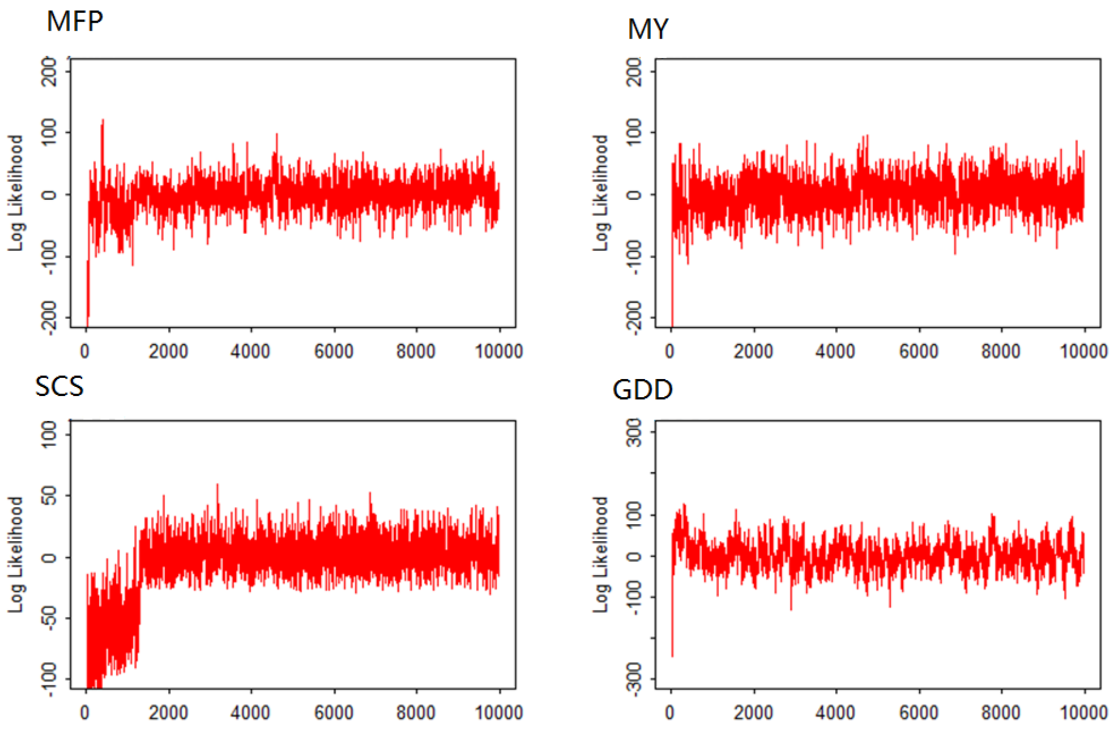
MFP
MY
SCS
GDD
Log Likelihood
0
2000
4000
6000
8000
10000

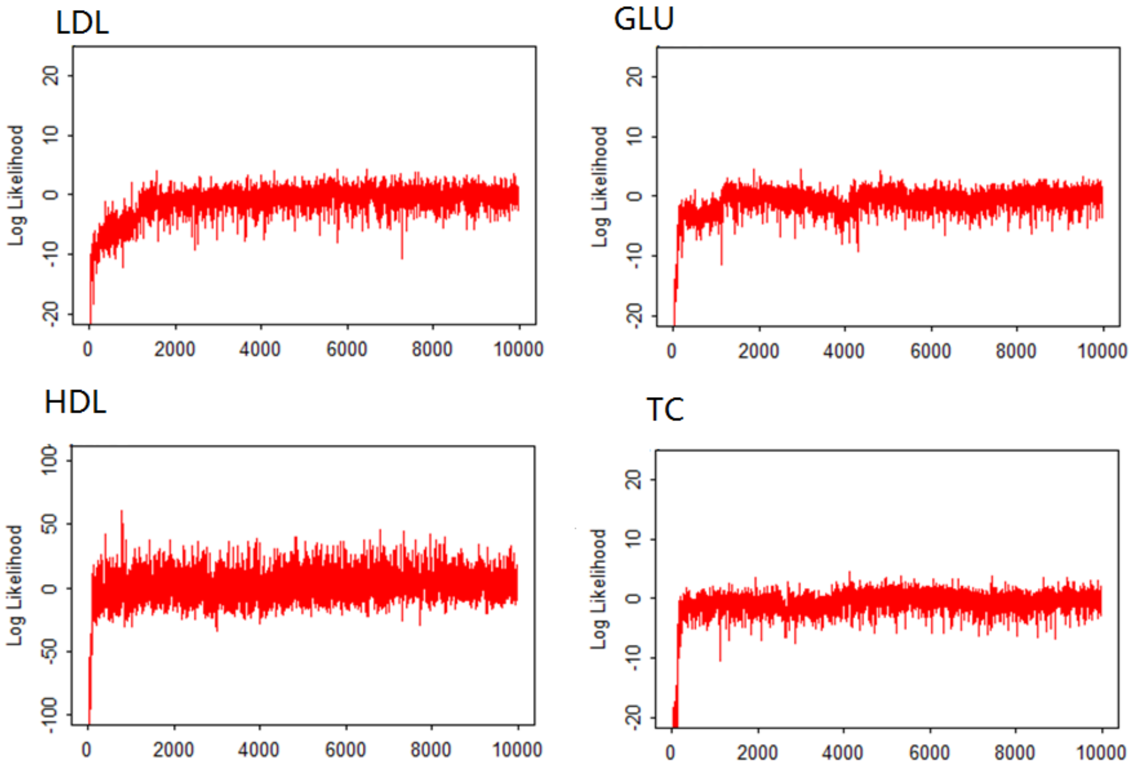
LDL
GLU
HDL
TC
Log Likelihood
0
2000
4000
6000
8000
10000

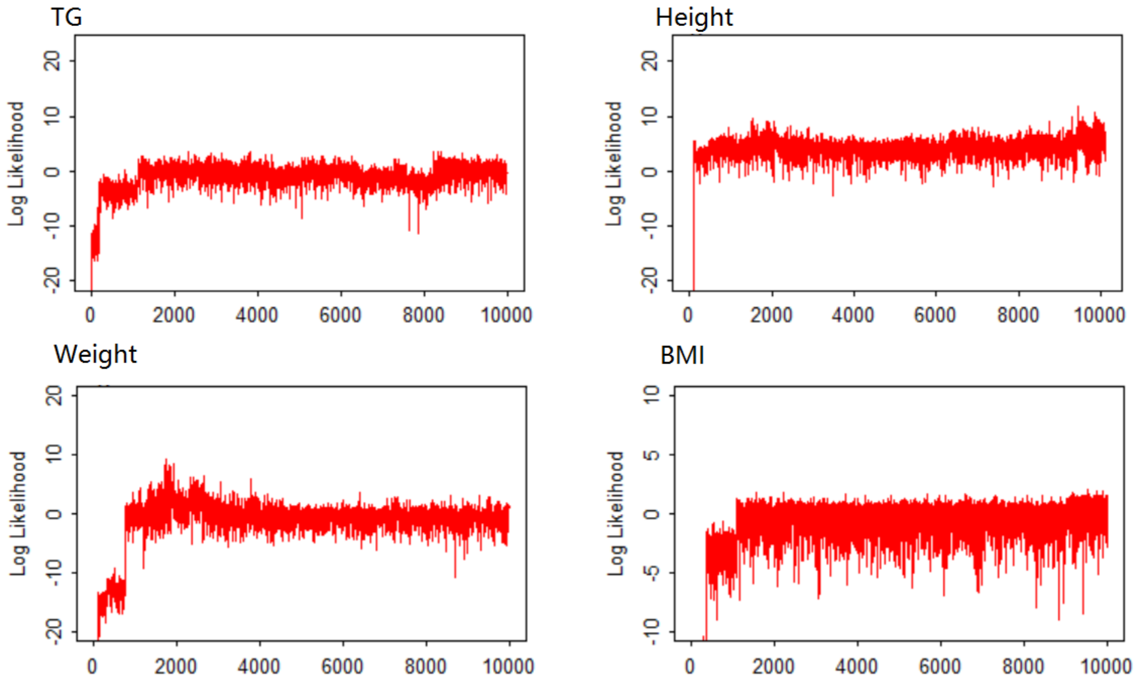

Supplementary Figure 8. Trace plots of the log posterior likelihood of DPR.MCMC in real data applications. For each of the twelve traits in the three GWAS data sets, we plot the log posterior likelihood versus the first 10,000 iterations (i.e. burn-in period) using the first cross-validation data. In each panel, the log posterior likelihood values were centered to have a median value of zero.

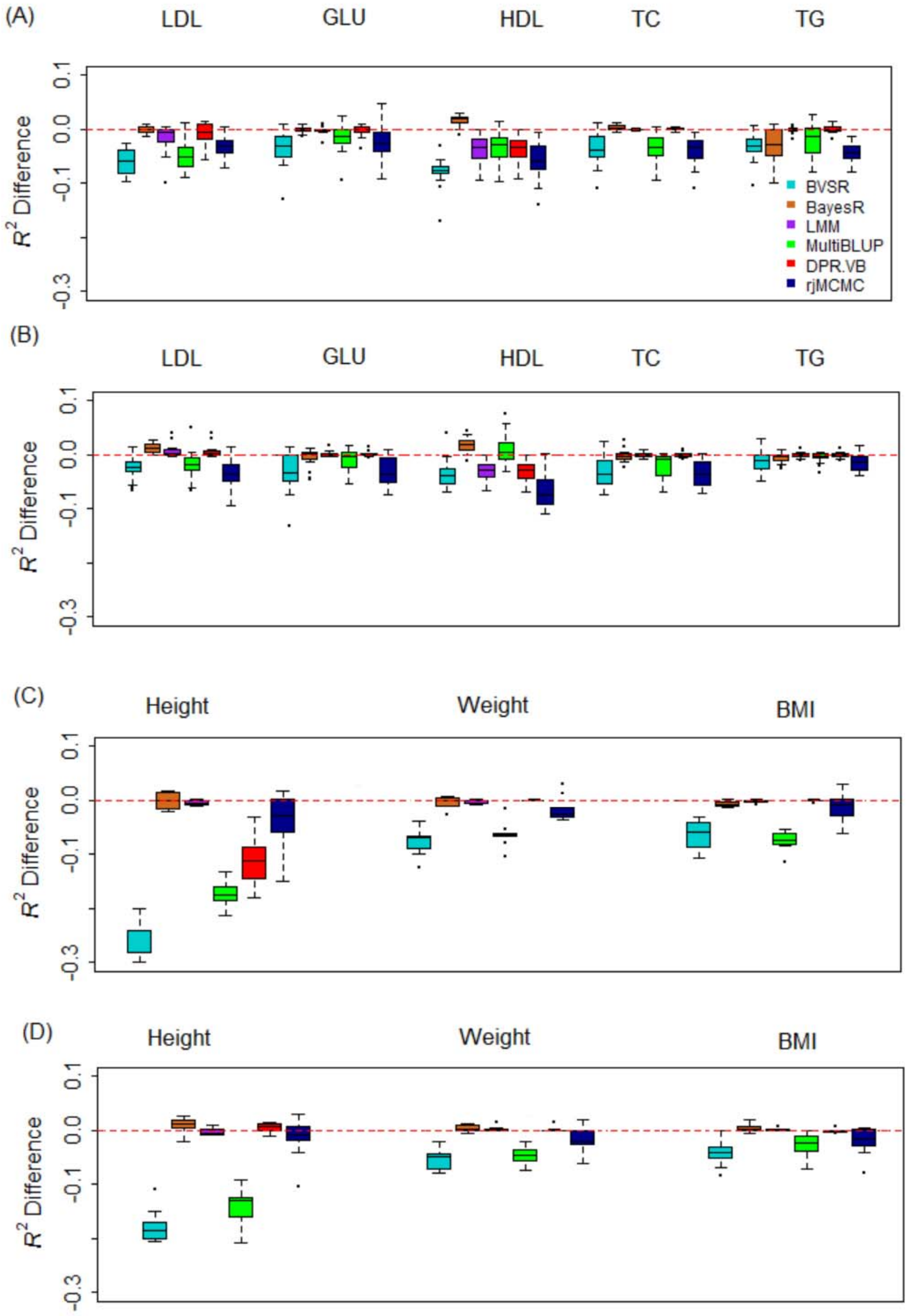

Supplementary Figure 9. Comparison of prediction performance of several methods with DPR.MCMC for eight traits in each of the two sub data sets of FHS. The two sub data sets D1 and D2 have the same sample size but different levels of relatedness

(individuals in D1 are more related to each other than those in D2). (A) The R^2 difference of five plasma traits (LDL, GLU, HDL, TC and TG) with respect to DPR.MCMC in the D1 and D2 sub data of FHS; (B) The R^2 difference of three anthropometric traits (Height, Weight and BMI) with respect to DPR.MCMC in the D1 and D2 sub data of FHS. For each box plot, the bottom and top of the box are the first and third quartiles, while the ends of whiskers represent either the lowest datum within 1.5 interquartile range of the lower quartile or the highest datum within 1.5 interquartile range of the upper quartile. FHS: Framingham heart study.

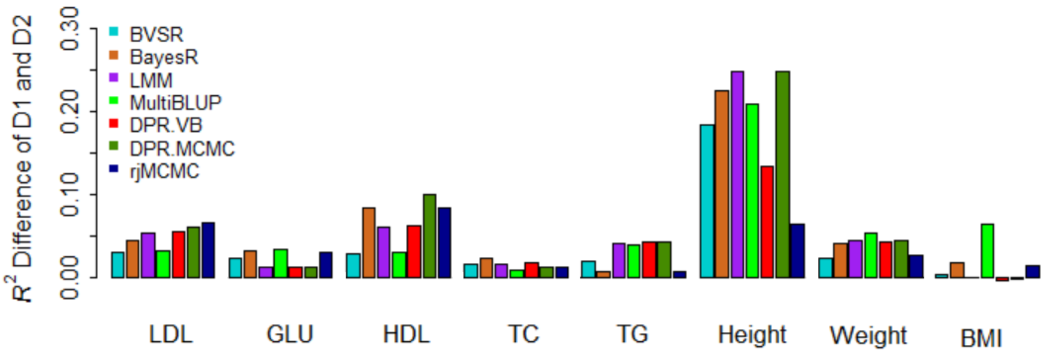

Supplementary Figure 10. Prediction performance of various methods are higher in a data with more related individuals (D1) than in a data with less related individuals (D2). The two data sets D1 and D2 from FHS have the same sample size but different levels of relatedness (individuals in D1 are more related to each other than those in D2). For each trait in the FHS data (x-axis), we first computed the median predictive R^2 across 20 replicates in D1 and D2 separately, and then contrast the difference between the two averaged predictive R^2 values in the two data sets (D1 minus D2; y-axis). Positive averaged predictive R^2 differences suggest that all methods have higher predictive performance in D1 versus D2. FHS: Framingham heart study.

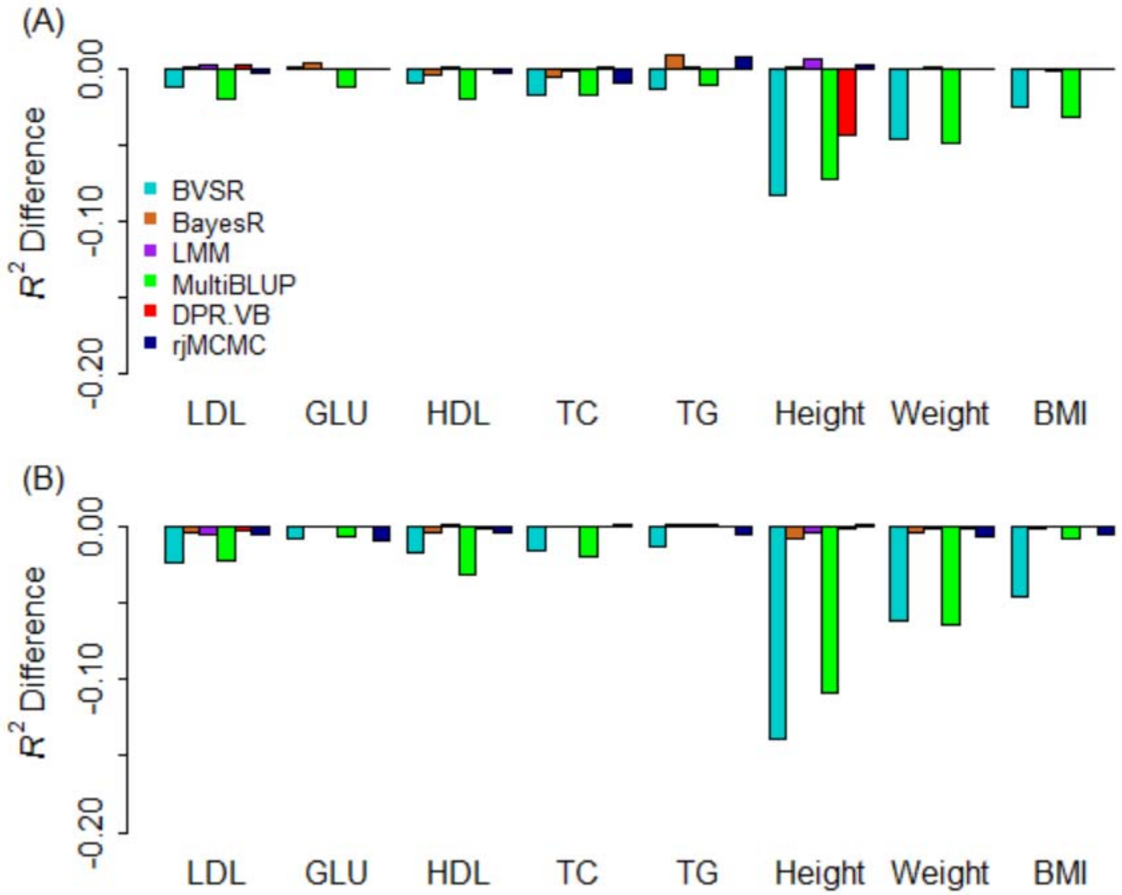

Supplementary Figure 11. Comparison of prediction performance of several methods with DPR.MCMC using cross-validation between the two sub data sets of FHS. The two sub data sets D1 and D2 have the same sample size but different levels of relatedness (individuals in D1 are more related to each other than those in D2). (A) Predictive R^2 difference of different methods in D1 using parameters inferred in D2. For DPR.MCMC, the R^2 is 0.024 for LDL, 0.012 for GLU, 0.021 for HDL, 0.022 for TC, 0.016 for TG, 0.131 for Height, 0.061 for Weight and 0.041 for BMI. (B) Predictive R^2 difference of different methods in D2 using parameters inferred in D1; For DPR.MCMC, the R^2 is 0.043 for LDL, 0.009 for GLU, 0.033 for HDL, 0.021 for TC, 0.015 for TG, 0.226 for Height, 0.083 for Weight and 0.058 for BMI. FHS: Framingham heart study.

Supplementary Table 1. Sampling variation of R^2 measured by standard deviation across Monte Carlo cross validation replicates for various methods in simulations and real data analysis.

		BVSR	rjMCMC	BayesR	LMM	MultiBLUP	DPR	
							VB	MCMC
**Simulations**								
PVE = 0.2								
I		0.019	0.019	0.020	0.019	0.019	0.019	0.019
II		0.016	0.016	0.016	0.015	0.015	0.016	0.016
III	10	0.017	0.017	0.019	0.018	0.018	0.017	0.017
	100	0.018	0.018	0.018	0.019	0.019	0.018	0.018
	1,000	0.015	0.015	0.015	0.016	0.016	0.015	0.015
IV	normal	0.023	0.023	0.023	0.023	0.023	0.023	0.023
	t	0.016	0.016	0.016	0.015	0.015	0.016	0.016
	Laplace	0.017	0.017	0.017	0.017	0.017	0.017	0.017
PVE = 0.5								
I		0.031	0.030	0.030	0.030	0.030	0.031	0.031
II		0.024	0.028	0.026	0.028	0.028	0.027	0.031
III	10	0.029	0.026	0.027	0.027	0.027	0.031	0.028
	100	0.031	0.031	0.031	0.030	0.030	0.031	0.031
	1,000	0.031	0.031	0.031	0.030	0.030	0.031	0.031
IV	normal	0.030	0.030	0.031	0.030	0.031	0.030	0.030
	t	0.025	0.025	0.025	0.027	0.026	0.025	0.025
	Laplace	0.023	0.023	0.023	0.024	0.024	0.024	0.024
PVE = 0.8								
I		0.027	0.029	0.029	0.028	0.028	0.029	0.029
II		0.028	0.022	0.022	0.022	0.022	0.022	0.024
III	10	0.022	0.024	0.022	0.023	0.023	0.024	0.024
	100	0.032	0.028	0.027	0.026	0.026	0.028	0.027
	1,000	0.035	0.030	0.030	0.030	0.030	0.030	0.030
IV	normal	0.030	0.028	0.028	0.028	0.028	0.028	0.028
	t	0.027	0.027	0.026	0.027	0.027	0.027	0.027
	Laplace	0.024	0.022	0.022	0.022	0.022	0.022	0.022
**Real data**								
Cattle								
	MFP	0.013	0.012	0.011	0.013	0.030	0.018	0.011
	MY	0.015	0.013	0.012	0.013	0.013	0.014	0.012
	SCS	0.019	0.020	0.018	0.018	0.016	0.022	0.017
Maize								
	GDD	0.013	0.011	0.012	0.010	0.014	0.013	0.012
FHS								
	LDL	0.013	0.013	0.032	0.014	0.033	0.014	0.012
	GLU	0.010	0.010	0.022	0.015	0.022	0.016	0.012
	HDL	0.010	0.021	0.029	0.015	0.067	0.018	0.019
	TC	0.011	0.014	0.019	0.009	0.020	0.016	0.015
	TG	0.008	0.014	0.018	0.020	0.022	0.011	0.014
	Height	0.032	0.047	0.051	0.045	0.048	0.050	0.050
	Weight	0.037	0.042	0.040	0.029	0.040	0.042	0.040
	BMI	0.034	0.038	0.036	0.035	0.036	0.041	0.039

Supplementary Table 2. Significant genes identified by DPR.MCMC for different diseases in the PrediXcan gene set analysis of WTCCC.

Disease	Gene	Chr	TSS	z score	p value	#SNPs	h^2	References
T1D	*LINC00240*[M,V]	6	26,988,232	5.73	9.78E-09	277	0.255	28
T1D	*ZNF165*[M,V]	6	28,048,753	7.40	1.40E-13	396	0.231	28
T1D	*ZNF192*[M,V]	6	28,109,716	6.80	1.04E-11	387	0.041	28
T1D	*TRIM3*[H,V]	6	30,080,883	-6.77	1.30E-11	13	0.089	28,29
T1D	*HCG18*[H,V]	6	30,294,927	-5.42	5.85E-08	9	0.468	29-33
T1D	*IER3*[H,V]	6	30,712,331	-7.07	1.60E-12	35	0.405	29-33
T1D	*DDR1*[H,V]	6	30,844,198	-7.31	2.76E-13	24	0.217	29-33
T1D	*VARS2*[H,V]	6	30,876,019	-5.05	4.34E-07	16	0.195	29-33
T1D	*MUC22*[H,V]	6	30,978,251	5.85	5.05E-09	148	0.155	29-33
T1D	*HCG22*[H,V]	6	31,021,227	-4.54	5.55E-06	177	0.719	29-33
T1D	*HLA-B*[H,V]	6	31,324,965	4.74	2.12E-06	153	0.579	29-33
T1D	*MICA*[H,V]	6	31,367,561	4.81	1.50E-06	114	0.157	29-33
T1D	*MICB*[H,V]	6	31,462,658	4.45	8.59E-06	66	0.620	29-33
T1D	*LST1*[H,V]	6	31,553,901	14.49	1.46E-47	42	0.377	29-33
T1D	*AGPAT1*[H,V]	6	32,145,873	-9.50	2.04E-21	13	0.046	29-33
T1D	*HLA-DRB5*[H,V]	6	32,498,064	-5.04	4.70E-07	28	0.741	29-33
T1D	*HLA-DQA2*[G]	6	32,709,119	18.85	2.99E-79	103	0.709	33
T1D	*HLA-DQB2*[H,V]	6	32,731,311	10.78	4.15E-27	119	0.778	33
T1D	*TAP2*[H,V]	6	32,806,599	-4.43	9.45E-06	111	0.815	33
T1D	*PSMB9*[H,V]	6	32,811,913	4.71	2.44E-06	120	0.205	33
T1D	*TAP1*[H,V]	6	32,821,755	8.60	7.70E-18	113	0.066	33
T1D	*HLA-DOA*[H,V]	6	32,977,389	-7.36	1.88E-13	55	0.152	33
T1D	*HLA-DPA1*[H,V]	6	33,048,552	6.80	1.04E-11	73	0.423	33,34
T1D	*HSD17B8*[H,V]	6	33,172,419	7.99	1.40E-15	46	0.194	33,34
T1D	*RPS26*[G]	12	56,435,637	5.93	2.97E-09	74	0.805	31
CD	*POU5F1*[H,V]	6	31,148,508	4.23	2.35E-05	260	0.526	31,35-39
CD	*LINC00481*[H,V]	6	31,169,695	4.47	7.70E-06	256	0.281	31,35-39
CD	*PTGER4*[G]	5	40,679,600	5.31	1.11E-07	292	0.182	40
CD	*AC091132.3*[V]	17	43,595,264	4.48	7.40E-06	24	0.557	35,37,41
CD	*PTPN2*[G]	18	12,884,337	-5.01	5.58E-07	194	0.260	31,37,40,41
CD	*STMN3*[V]	20	62,284,780	-4.43	9.38E-06	96	0.277	37
RA	*PANK4*[V]	1	2,458,039	4.39	1.13E-05	64	0.126	42-44
RA	*HLA-G*[G]	6	29,794,744	4.54	5.57E-06	64	0.459	43,45-57
RA	*TRIM26*[V]	6	30,181,204	-5.85	4.80E-09	12	0.044	45
RA	*IER3*[V]	6	30,712,331	-5.23	1.72E-07	35	0.405	43,45-57
RA	*HLA-DRB5*[V]	6	32,498,064	-6.84	8.11E-12	28	0.741	43,45-57

RA	*HLA-DQA2*[G]	6	32,709,119	9.51	1.82E-21	103	0.709	52,58
RA	*HLA-DQB2*[V]	6	32,731,311	9.38	6.88E-21	119	0.778	43,45-57

The table also lists the disease name, gene id, chromosome number, transcription start site (TSS), association strength (z score, p value), the number of SNPs in each gene set test, estimated SNP heritability (h^2, from GEMMA), and references that support the identified association. T1D: type 1 diabetes, CD: Crohn's disease, RA: rheumatoid arthritis. H indicates Human leukocyte antigen (HLA) region genes on chromosome 6, M indicates major histocompatibility complex (MHC) region, G indicates genes previously identified to be associated with diseases in the NHGRI GWAS catalog, V indicates the vicinity of a reported gene. h^2 is the estimator of heritability using linear mixed models in GEMMA.

Supplementary References

1. Zhou, X., Carbonetto, P., & Stephens, M. Polygenic modeling with Bayesian sparse linear mixed models. *PLoS Genet.* **9,** e1003264 (2013).
2. Yang, J. *et al.* Common SNPs explain a large proportion of the heritability for human height. *Nat. Genet.* **42,** 565-569 (2010).
3. Moser, G. *et al.* Simultaneous Discovery, Estimation and Prediction Analysis of Complex Traits Using a Bayesian Mixture Model. *PLoS Genet.* **11,** e1004969 (2015).
4. Robert, C., & Casella, G. *Monte Carlo statistical methods* (Second ed.). New York: Springer (2002).
5. Gelman, A. Parameterization and Bayesian Modeling. *J. Am. Stat. Assoc.* **99,** 537-545 (2004).
6. Visscher, P. M., Hill, W. G., & Wray, N. R. Heritability in the genomics era--concepts and misconceptions. *Nat. Rev. Genet.* **9,** 255-266 (2008).
7. de los Campos, G., Sorensen, D., & Gianola, D. Genomic heritability: what is it? *PLoS Genet.* **11,** e1005048 (2015).
8. Zhou, X., & Stephens, M. Genome-wide efficient mixed-model analysis for association studies. *Nat. Genet.* **44,** 821-824 (2012).
9. Lippert, C. *et al.* FaST linear mixed models for genome-wide association studies. *Nat. Methods* **8,** 833-835 (2011).
10. Levine, R. A., & Casella, G. Optimizing random scan Gibbs samplers. *J. Multivariate Anal.* **97,** 2071-2100 (2006).
11. Levine, R. A., Yu, Z., Hanley, W. G., & Nitao, J. J. Implementing random scan Gibbs samplers. *Comput Stat* **20,** 177-196 (2005).
12. Blei, D. M., & Jordan, M. I. Variational inference for Dirichlet process mixtures. *Bayesian. Anal.* **1,** 121-143 (2006).
13. Ishwaran, H., & James, L. F. Approximate Dirichlet Process Computing in Finite Normal Mixtures. *J. Comput. Graph. Statist.* **11,** 508-532 (2002).
14. Ishwaran, H., & James, L. F. Gibbs sampling methods for stick-breaking priors. *J. Am. Stat. Assoc.* **96,** (2001).
15. Gelman, A. *et al. Bayesian Data Analysis* (Third ed.). New York: Chapman & Hall/CRC (2013).
16. Spiegelhalter, D. J., Best, N. G., Carlin, B. P., & Van Der Linde, A. Bayesian measures of model complexity and fit. *J. R. Stat. Soc. Ser. B.* **64,** 583-639 (2002).
17. Gelman, A., Hwang, J., & Vehtari, A. Understanding predictive information criteria for Bayesian models. *Stat. Comput.* **24,** 997-1016 (2014).
18. Brooks, S. Markov chain Monte Carlo method and its application. *Journal of the royal statistical society: series D (the Statistician)* **47,** 69-100 (1998).
19. Hastie, T., Tibshirani, R., & Friedman, J. H. *The elements of statistical learning: data mining, inference, and prediction*. New York, NY: Springer (2009).
20. Bishop, C. M. *Pattern recognition and machine learning*. New York: Springer (2006).
21. Jordan, M. I., Ghahramani, Z., Jaakkola, T. S., & Saul, L. K. An introduction to variational methods for graphical models. *Mach. Learn.* **37,** 183-233 (1999).
22. Grimmer, J. An Introduction to Bayesian Inference via Variational Approximations. *Pol. Anal.* **19,** 32-47 (2011).

23. Ormerod, J. T., & Wand, M. Explaining variational approximations. *Am. Stat.* **64,** 140-153 (2010).

24. Pham, T. H., Ormerod, J. T., & Wand, M. P. Mean field variational Bayesian inference for nonparametric regression with measurement error. *Comput. Stat. Data Anal.* **68,** 375-387 (2013).

25. Wand, M. P., Ormerod, J. T., Padoan, S. A., & Fuhrwirth, R. Mean field variational Bayes for elaborate distributions. *Bayesian. Anal.* **6,** 847-900 (2011).

26. Blei, D. M., Kucukelbir, A., & McAuliffe, J. D. Variational inference: A review for statisticians. *J. Am. Stat. Assoc. (in press), Preprint at https://arxiv.org/abs/1601.00670* (2017).

27. Wang, C., & Blei, D. M. Variational inference in nonconjugate models. *J. Mach. Learn. Res.* **14,** 1005-1031 (2013).

28. DIAbetes Genetics Replication And Meta-analysis (DIAGRAM) Consortium *et al.* Genome-wide trans-ancestry meta-analysis provides insight into the genetic architecture of type 2 diabetes susceptibility. *Nat. Genet.* **46,** 234-244 (2014).

29. Barrett, J. C. *et al.* Genome-wide association study and meta-analysis find that over 40 loci affect risk of type 1 diabetes. *Nat. Genet.* **41,** 703-707 (2009).

30. Cooper, J. D. *et al.* Meta-analysis of genome-wide association study data identifies additional type 1 diabetes risk loci. *Nat. Genet.* **40,** 1399-1401 (2008).

31. The Wellcome Trust Case Control Consortium. Genome-wide association study of 14,000 cases of seven common diseases and 3,000 shared controls. *Nature* **447,** 661-678 (2007).

32. Hakonarson, H. *et al.* A genome-wide association study identifies KIAA0350 as a type 1 diabetes gene. *Nature* **448,** 591-594 (2007).

33. Perry, J. R. *et al.* Stratifying type 2 diabetes cases by BMI identifies genetic risk variants in LAMA1 and enrichment for risk variants in lean compared to obese cases. *PLoS Genet.* **8,** e1002741 (2012).

34. Lin, H. *et al.* Novel susceptibility genes associated with diabetic cataract in a Taiwanese population. *Ophthalmic Genet.* **34,** 35-42 (2013).

35. Yamazaki, K. *et al.* A genome-wide association study identifies 2 susceptibility loci for Crohn's disease in a Japanese population. *Gastroenterology* **144,** 781-788 (2013).

36. Jostins, L. *et al.* Host-microbe interactions have shaped the genetic architecture of inflammatory bowel disease. *Nature* **491,** 119-124 (2012).

37. Franke, A. *et al.* Genome-wide meta-analysis increases to 71 the number of confirmed Crohn's disease susceptibility loci. *Nat. Genet.* **42,** 1118-1125 (2010).

38. Julià, A. *et al.* A genome-wide association study on a southern European population identifies a new Crohn's disease susceptibility locus at RBX1-EP300. *Gut* **62,** 1440-1445 (2013).

39. Yang, S. K. *et al.* Genome-wide association study of Crohn's disease in Koreans revealed three new susceptibility loci and common attributes of genetic susceptibility across ethnic populations. *Gut* **63,** 80-87 (2014).

40. Parkes, M. *et al.* Sequence variants in the autophagy gene IRGM and multiple other replicating loci contribute to Crohn's disease susceptibility. *Nat. Genet.* **39,** 830-832 (2007).

41. Barrett, J. C. *et al.* Genome-wide association defines more than 30 distinct susceptibility loci for Crohn's disease. *Nat. Genet.* **40,** 955-962 (2008).
42. Orozco, G. *et al.* Novel Rheumatoid Arthritis Susceptibility Locus at 22q12 Identified in an Extended UK Genome-Wide Association Study. *Arthritis Rheumatol.* **66,** 24-30 (2014).
43. Stahl, E. A. *et al.* Genome-wide association study meta-analysis identifies seven new rheumatoid arthritis risk loci. *Nat. Genet.* **42,** 508-514 (2010).
44. Raychaudhuri, S. *et al.* Common variants at CD40 and other loci confer risk of rheumatoid arthritis. *Nat. Genet.* **40,** 1216-1223 (2008).
45. Eleftherohorinou, H., Hoggart, C. J., Wright, V. J., Levin, M., & Coin, L. J. Pathway-driven gene stability selection of two rheumatoid arthritis GWAS identifies and validates new susceptibility genes in receptor mediated signalling pathways. *Hum. Mol. Genet.* **20,** 3494-3506 (2011).
46. Hüffmeier, U. *et al.* Common variants at TRAF3IP2 are associated with susceptibility to psoriatic arthritis and psoriasis. *Nat. Genet.* **42,** 996-999 (2010).
47. Bossini-Castillo, L. *et al.* A genome-wide association study of rheumatoid arthritis without antibodies against citrullinated peptides. *Ann. Rheum. Dis.* annrheumdis-2013-204591 (2014).
48. Hu, H.-J. *et al.* Common variants at the promoter region of the APOM confer a risk of rheumatoid arthritis. *Exp. Mol. Med.* **43,** 613-621 (2011).
49. Terao, C. *et al.* The human AIRE gene at chromosome 21q22 is a genetic determinant for the predisposition to rheumatoid arthritis in Japanese population. *Hum. Mol. Genet.* **20,** 2680-2685 (2011).
50. Orozco, G. *et al.* Novel Rheumatoid Arthritis Susceptibility Locus at 22q12 Identified in an Extended UK Genome‐Wide Association Study. *Arthritis Rheumatol.* **66,** 24-30 (2014).
51. Behrens, E. M. *et al.* Association of the TRAF1–C5 locus on chromosome 9 with juvenile idiopathic arthritis. *Arthritis Rheum.* **58,** 2206-2207 (2008).
52. Nakajima, M. *et al.* New sequence variants in HLA class II/III region associated with susceptibility to knee osteoarthritis identified by genome-wide association study. *PLoS ONE* **5,** e9723 (2010).
53. Okada, Y. *et al.* Genetics of rheumatoid arthritis contributes to biology and drug discovery. *Nature* **506,** 376-381 (2014).
54. Jiang, L. *et al.* Novel risk loci for rheumatoid arthritis in Han Chinese and congruence with risk variants in Europeans. *Arthritis Rheumatol.* **66,** 1121-1132 (2014).
55. Padyukov, L. *et al.* A genome-wide association study suggests contrasting associations in ACPA-positive versus ACPA-negative rheumatoid arthritis. *Ann. Rheum. Dis.* (2010).
56. Plenge, R. M. *et al.* TRAF1–C5 as a risk locus for rheumatoid arthritis—a genomewide study. *N. Engl. J. Med.* **357,** 1199-1209 (2007).
57. Freudenberg, J. *et al.* Genome-wide association study of rheumatoid arthritis in Koreans: Population-specific loci as well as overlap with European susceptibility loci. *Arthritis Rheum.* **63,** 884-893 (2011).

58. Julia, A. *et al.* Genome‐wide association study of rheumatoid arthritis in the Spanish population: KLF12 as a risk locus for rheumatoid arthritis susceptibility. *Arthritis Rheum.* **58,** 2275-2286 (2008).
